# Transdifferentiation of Fast Skeletal Muscle Into Functional Endothelium in Vivo by Transcription Factor Etv2

**DOI:** 10.1371/journal.pbio.1001590

**Published:** 2013-06-18

**Authors:** Matthew B. Veldman, Chengjian Zhao, Gustavo A. Gomez, Anne G. Lindgren, Haigen Huang, Hanshuo Yang, Shaohua Yao, Benjamin L. Martin, David Kimelman, Shuo Lin

**Affiliations:** 1 State Key Laboratory of Biotherapy and Cancer Center, West China Hospital, West China Medical School, Sichuan University, Chengdu, Sichuan, People's Republic of China; 2 Department of Molecular, Cell and Developmental Biology, University of California–Los Angeles, Los Angeles, California, United States of America; 3 Department of Biochemistry and Cell Biology, Stony Brook University, Stony Brook, New York, United States of America; 4 Department of Biochemistry, University of Washington, Seattle, Washington, United States of America; The Wellcome Trust Sanger Institute, United Kingdom

## Abstract

Etsrp/Etv2 (Etv2) is an evolutionarily conserved master regulator of vascular development in vertebrates. Etv2 deficiency prevents the proper specification of the endothelial cell lineage, while its overexpression causes expansion of the endothelial cell lineage in the early embryo or in embryonic stem cells. We hypothesized that Etv2 alone is capable of transdifferentiating later somatic cells into endothelial cells. Using heat shock inducible Etv2 transgenic zebrafish, we demonstrate that Etv2 expression alone is sufficient to transdifferentiate fast skeletal muscle cells into functional blood vessels. Following heat treatment, fast skeletal muscle cells turn on vascular genes and repress muscle genes. Time-lapse imaging clearly shows that muscle cells turn on vascular gene expression, undergo dramatic morphological changes, and integrate into the existing vascular network. Lineage tracing and immunostaining confirm that fast skeletal muscle cells are the source of these newly generated vessels. Microangiography and observed blood flow demonstrated that this new vasculature is capable of supporting circulation. Using pharmacological, transgenic, and morpholino approaches, we further establish that the canonical Wnt pathway is important for induction of the transdifferentiation process, whereas the VEGF pathway provides a maturation signal for the endothelial fate. Additionally, overexpression of Etv2 in mammalian myoblast cells, but not in other cell types examined, induced expression of vascular genes. We have demonstrated in zebrafish that expression of Etv2 alone is sufficient to transdifferentiate fast skeletal muscle into functional endothelial cells in vivo. Given the evolutionarily conserved function of this transcription factor and the responsiveness of mammalian myoblasts to Etv2, it is likely that mammalian muscle cells will respond similarly.

## Abstract 2

Etv2, a master regulator of endothelial cell fate, can induce fast skeletal muscle cells to transdifferentiate into endothelial cells in the zebrafish embryo.

## Author Summary

The endothelial cell is a specialized cell type that lines blood vessels. These cells are involved in normal cardiovascular function and become damaged in cardiovascular disease states such as atherosclerosis and stroke. We have discovered that developing muscle cells in the zebrafish embryo can be converted into endothelial cells by the expression of a transcription factor called Etv2. Etv2 normally functions during embryonic development to specify blood and blood vessels. When expressed in muscle cells, Etv2 induces the expression of genes that are normally expressed in endothelial cells; it also represses muscle gene expression. On expressing Etv2, muscle cells change shape and go on to form lumenized blood vessels that connect to the existing circulatory system and support blood flow. The Wnt and VEGF signaling pathways are required for this fate transformation. Our results suggest that muscle cells may be a viable source for the de novo generation of endothelial cells for use in transplantation therapies and they highlight signalling pathways that might be manipulated to improve the efficiency of this process in mammalian cells.

## Introduction

The ETS family transcription factor Etv2 (also known as Etsrp or ER71) is an evolutionarily conserved early mediator of blood vessel and blood cell development. In zebrafish, Etv2 is expressed in the lateral plate mesoderm at early somitogenesis stages and defines the first population of angioblasts to arise in the embryo [1]. Knocking down this gene using morpholino antisense oligonucleotides or a genetic mutation causes defects in vasculogenesis, angiogenesis, and arteriovenus specification accompanied by decreased expression of many vascular genes including transcription factors *fli1a* and *tal1* (also known as *scl*), and *kdrl* (formerly *flk1*), a vascular endothelial growth factor (VEGF) receptor [1],[2]. Similarly in *Xenopus*, blocking ETV2 expression decreases the expression of critical vascular genes such as *flk1*, *aplnr*, *erg*, and *ve-cadherin* [3],[4]. In mice, Etv2 is expressed in the early vascular and hematopoietic structures of the yolk sac and embryo proper [5]. Lineage tracing using an *Etv2:Cre* line demonstrated that *Etv2* positive cells give rise to the adult endothelium and blood cells [6]. The *Etv2* knockout mouse dies around embryonic day 10 due to cardiovascular defects and also has significantly reduced levels of Kdr (formerly Flk1) [5],[7]. Chimeric mouse analysis with *Etv2* knockout cells demonstrated that these cells are specifically not capable of contributing to the blood or endothelial cell lineages [8]. These studies demonstrate that Etv2 is necessary for vascular development in multiple species.

The very early expression of Etv2 and its necessity in vascular development suggest that it may be near the top of the transcriptional hierarchy regulating endothelial cell specification. Overexpression of Etv2 in mouse embryonic stem cells induces the expression of many hematopoietic and vascular genes and increases the yield of these cells when differentiated [5],[9]. A combined FOX:ETS binding site bound by Etv2 is found near most described vascular genes in the mouse genome [10]. In vivo, overexpression of ETV2 in *Xenopus* causes ectopic expression vascular genes including *flk1* [3],[4]. Similarly, in zebrafish, Etv2 overexpression induces the ectopic expression of vascular genes *kdrl*, *fli1a*, and *tal1* amongst hundreds of others recently defined by microarray and RNA-seq studies [11]–[13]. Although these studies have demonstrated that Etv2 can induce expression of endothelial genes in multiple model systems, no one has directly tested if these cells can form functional vasculature in vivo.

A group of recent studies have established that Etv2 is also a critical factor regulating the balance between endocardial versus myocardial cell fates within the anterior lateral plate mesoderm that forms the heart. In zebrafish, knockdown of *etv2* and *tal1* results in rostral expansion of myocardial precursor cells at the expense of endothelial and primitive myeloid cells [14]. In fact, loss of *etv2* alone is sufficient for expansion of myocardium [15]. Conversely, overexpression of *etv2* and *tal1* repress myocardial fates while expanding vascular and primitive myeloid fates [14]. In mouse ES cell culture overexpression of Etv2 was found to not only bias cells towards endothelial and hematopoietic fates but also to repress myocardial fates [8]. Loss of Etv2 enhanced smooth muscle cell and cardiomyocyte generation in these culture models. In vivo, the myocardial lineage in Etv2 knockout mice is significantly expanded [6]. Using fluorescent activated cell sorting and gene expression analysis from *Etv2:EYFP* transgenic mouse embryos, Rasmussen et al. [6] found hematopoietic and endothelial genes*Tal1* and *Fli1* as well as endocardial genes *Nfatc1* and *Sox17* among the highly expressed genes in this cell population. When Etv2 was knocked out in the EYFP population in vivo, elevated expression of myocardial genes such as *Nkx2–5*, *Gata4*, and *Tbx5* as well as skeletal muscle and smooth muscle genes were noted. In total, all of the studies described above place Etv2 at the top of the genetic hierarchy that defines the balance of endothelial and hematopoietic lineages from cardiac and myogenic lineages in the embryonic mesoderm.

The regenerative medicine field is very interested in the possibility of generating patient-specific replacement tissues or organs from adult or induced pluripotent stem cells or other cellular sources. Sometimes complex drug and growth factor treatment protocols are necessary to achieve the desired result. However, overexpression of one or a few key developmental transcription factors is often all that is needed to drive a cell from one fate to another. Given Etv2's place atop the genetic hierarchy for the endothelial cell lineage, we were interested in testing the ability of this transcription factor to induce blood vessel formation as a possible treatment for vascular disease.

In this study we used zebrafish as a model to demonstrate that overexpression of Etv2 by itself is sufficient to generate functional ectopic blood vessels in vivo. Intriguingly, we found that the additional endothelial cells derived from fast skeletal muscle, revealing an unknown potential for embryonic muscle cells to convert to vasculature. In contrast, two other transcription factors downstream of Etv2 but still near the top of the endothelial lineage, Fli1a and Tal1, are not sufficient to induce blood vessels from muscle. Using time-lapse microscopy of transgenic fish we show that fast muscle fibers change morphology, repress muscle specific genes, and induce vascular genes. Muscle fiber-derived endothelial cells initially change shape, forming large vacuoles and retracting from their long fiber-like shape. They then extend processes and contact other transdifferentiating cells or established endothelial cells, and form patent vessels that support blood flow. To identify signaling pathways supporting this process, we performed a chemical compound screen. We found that precise levels of Wnt signaling are necessary for the efficient induction of vascular genes downstream of Etv2 in transdifferentiating muscle. Additionally, we found that VEGF signaling is necessary for the morphological differentiation of these cells into vessels but is dispensable for the induction of vascular genes by Etv2. Our findings suggest that activation of Wnt signaling coupled with Etv2 expression may be a viable method for inducing transdifferentiation of muscle precursor cells into endothelial cells for therapeutic purposes.

## Results

### Overexpression of Etv2 Induces Ectopic Vascular Gene Expression and Represses Muscle

**Figure 1:**
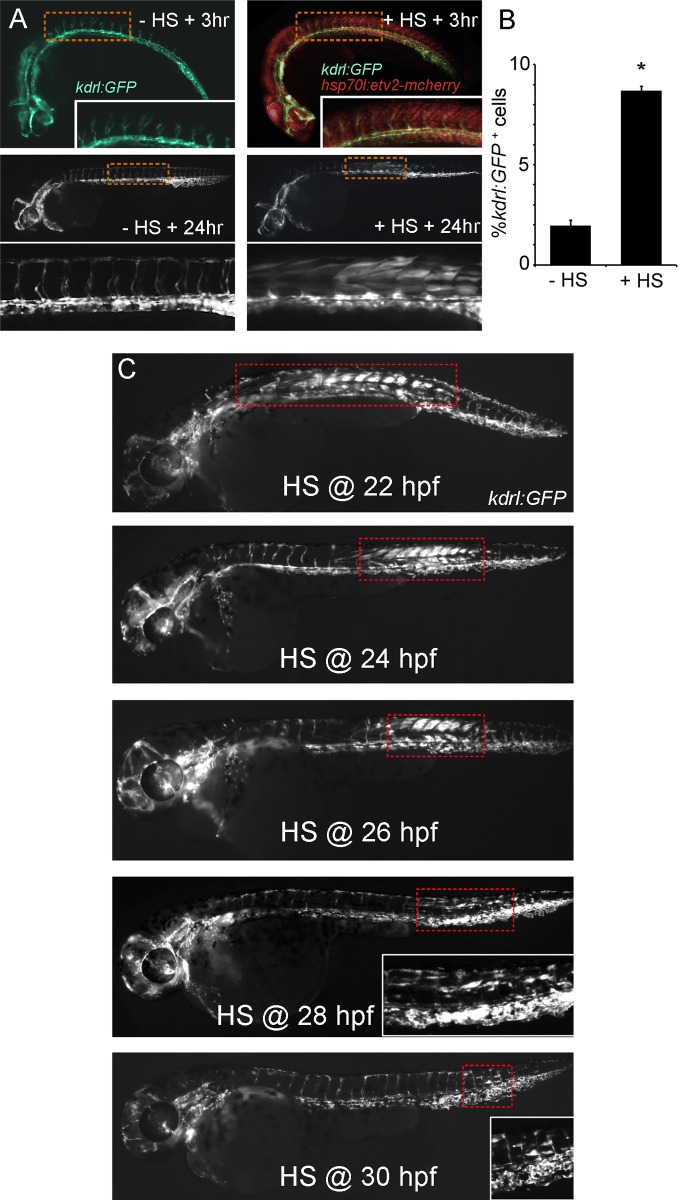
Ubiquitous Etv2 expression induces ectopic vascular gene expression in the trunk of 48 h zebrafish embryos. (A) *hsp70l:etv2/kdrl:GFP* embryo at 27 h postfertilization (hpf) (high magnification view of trunk inset) and 48 hpf exhibit normal vascular-specific GFP expression and no Etv2-mCherry when not treated with heat shock (−HS+3 h and +24 h). Embryos heat shocked at 24 hpf exhibit normal vascular GFP expression and strong ubiquitous nuclear Etv2-mCherry expression at 27 hpf (+HS+3 h). By 24 h post–heat shock (48 hpf) robust ectopic GFP expression is present in the trunk. (B) Flow cytometric analysis of single cells isolated from three separate clutches of *hsp70l:etv2/kdrl:GFP* embryos treated with or without heat shock at 24 hpf and analyzed at 48 hpf. Ectopic Etv2 expression causes the percentage of GFP^+^ cells to increase from ∼2% to ∼8% of the total. *T-test* (*) *p*<0.05. (C) Response to Etv2 overexpression is developmentally restricted. *Hsp70l:etv2/kdrl:GFP* embryos heat shocked (HS) at 22, 24, 26, 28, or 30 hpf exhibit decreasing numbers of GFP^+^ cells and a shift from anterior trunk to tail. Heat shock after 30 hpf did not cause ectopic GFP^+^ cells. Dashed boxes highlight the Etv2 responsive cell populations.

In zebrafish embryos, overexpression of Etv2 by mRNA injection into the single-cell embryo induces the ectopic expression of endothelial and hematopoietic genes but also causes gastrulation defects. To bypass this early embryonic toxicity and examine the effects of Etv2 overexpression in later developmental and adult stages, we generated transgenic fish in which an Etv2-mCherry fusion protein is under the control of the *hsp70l* promoter (named *hsp70l:etv2* hereafter). We used the Etv2-mCherry fusion so we could visualize ectopic Etv2 expression directly. We tested the fusion protein for functionality by mRNA injection into *kdrl:GFP* zebrafish embryos and found that it was as potent as untagged Etv2 at inducing ectopic GFP expression (unpublished data). We confirmed the function of the new transgene by heat shocking double transgenic embryos (*hsp70l:etv2* crossed to *kdrl:GFP*) at dome stage and examining them for ectopic GFP expression. Similar to mRNA-injected embryos, *hsp70l:etv2*/*kdrl:GFP* embryos exhibit robust GFP expression (Figure S1). We also noted nuclear mCherry expression appearing 1 h post–heat shock (Figure S1). To test the developmental window in which cells are competent to respond to Etv2 overexpression, we performed a heat shock time course with *hsp70l:etv2*/*kdrl:GFP* embryos and looked for ectopic GFP expression. As expected, tissues appear to lose the capacity to respond to Etv2 as development progresses. However, we discovered robust ectopic GFP expression at 48 h postfertilization (hpf) restricted mainly to the trunk and tail of embryos that were heat shocked between 24 hpf and 30 hpf (Figure 1A and C). During this time period, responding cells progressed in an anterior to posterior wave with only a few cells responding in the tail by 30 hpf heat shock (Figure 1C). Etv2-mCherry was visible 1 h post–heat shock, peaked at 3 h post–heat shock, and disappeared between 6 and 8 h post–heat shock (Figure 1A and unpublished data). We measured the time course of Etv2-mCherry expression following heat shock by quantitative RT-PCR (qPCR) and found very high expression 4 h post–heat shock with much less but still elevated expression at 8 and 16 h post–heat shock (Figure S2). Ectopic *kdrl:GFP* expression initiated around 8 h post–heat shock and was very robust by 24 h post–heat shock (Figures 1A and S3). Consistently, all embryos that exhibited heat shock–induced Etv2 had ectopic *kdrl:GFP* expression. Quantification of GFP^+^ cells by flow cytometry comparing heat shock versus nonshocked embryos demonstrated a greater than 4-fold increase in *kdrl:GFP* ^+^ cells (Figure 1B).

**Figure 2:**
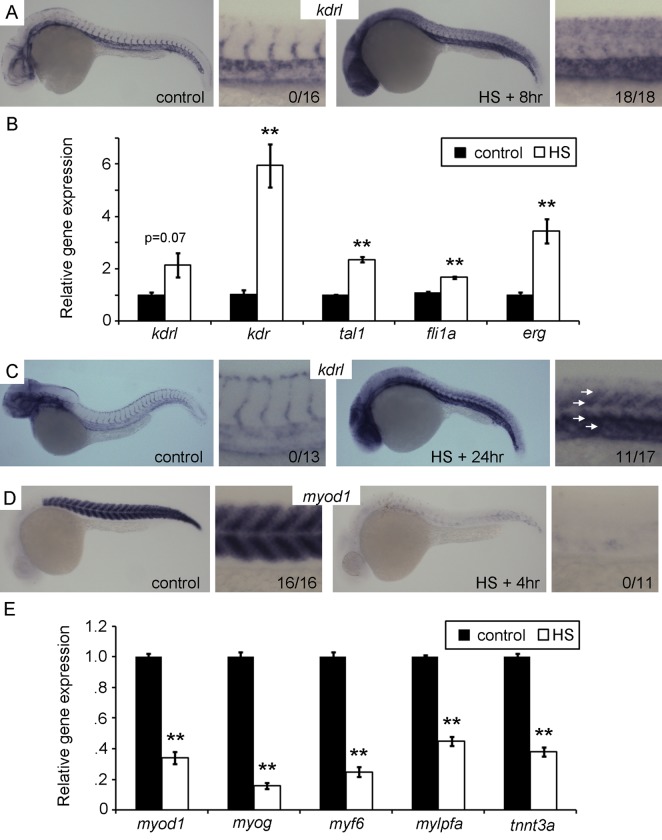
Etv2 overexpression induces vascular gene expression and represses muscle gene expression. (A and B) Vascular genes are induced 8 h post-HS. (A) In situ hybridization (ISH) for *kdrl* demonstrates broad ectopic expression including in the trunk following HS, but normal vascular restricted expression in control embryos. The numbers in the higher magnification views represent the number of embryos exhibiting ectopic expression over the number observed. (B) Quantitiative RT-PCR (qPCR) for *kdrl*, *kdr*, *tal1*, *fli1a*, and *erg* 8 h post-HS versus non–heat shocked controls. (C) ISH for *kdrl* at 24 h post-HS demonstrates increase expression in the trunk (white arrows). The apparent age discrepancy between the control and the HS+24 h embryo is due to developmental delay caused by Etv2 overexpression. (D and E) Muscle genes are repressed by Etv2 overexpression. (D) ISH for *myod1* demonstrating near complete loss of expression 4 h post-HS. The numbers in the higher magnification views represent the number of embryos exhibiting normal expression levels. (E) qPCR for muscle genes *myod1*, *myog*, *myf6*, *mylpfa*, and *tnnt3a* shows significantly decreased expression 4 h post–heat shock. qPCR was performed on three separate clutches. (**) *p*<0.01, *t* test versus non–heat shocked control.

The induction of *kdrl:GFP* by Etv2 suggested that nonendothelial cells were taking on an endothelial fate. To extend this finding we examined the expression of a panel of five genes—*kdrl*, *fli1a*, *tal1*, *erg*, and *kdr*—previously found to regulate vascular development in zebrafish [2],[16]–[22]. *Kdrl* and *kdr* are VEGF receptors, while *fli1a*, *tal1*, and *erg* are transcription factors necessary for successful vascular development. Initially, we took advantage of two transgenic lines, *fli1a:EGFP* [23] and *tal1:GFP*, to examined if the Etv2 response was similar to *kdrl:GFP*. A *tal1:GFP* transgenic line that faithfully recapitulates the pattern of *tal1* expression was previously identified in the lab using an enhancer trap approach (unpublished results). Both transgenic reporters displayed ectopic expression in the trunk following heat shock expression of Etv2, suggesting a broad genetic response was occurring (Figure S3). To test if endogenous gene induction was occurring, we performed qPCR and whole mount in situ hybridization (ISH) for the previously mentioned genes. By 8 h post–heat shock, ectopic expression of *kdrl* (Figure 2A), *fli1a*, *tal1*, *erg*, and *kdr* (Figure S4) was detected in the trunk as well as more broadly in the embryo by ISH. qPCR measurements at this timepoint confirms increased expression of these vascular genes (Figure 2B), although *kdrl* did not quite reach statistical significance (*p* = 0.07). Surprisingly, *kdrl* expression was broad at 8 h post–heat shock and not restricted to the trunk as seen with the *kdrl:GFP* transgene at 24 h. Therefore we performed ISH on 24 h post–heat shock embryos for *kdrl* and found expression remained fairly broad but was enriched in the trunk (Figure 2C). This demonstrates that an initial broad response within the embryo is resolving to the trunk region by 24 h post–heat shock.

The appearance of the ectopic vascular gene expression in the trunk occurred in cells reminiscent of muscle fibers by location and morphology. We reasoned that if these cells were truly switching fates they would have decreased expression of muscle genes. Therefore, we assayed five skeletal muscle–specific genes for changes in expression following heat shock. *Myod1*, *myog*, and *myf6* are bHLH transcription factors necessary for muscle development and strongly expressed at the time point examined. *Mylpfa* and *tnnt3a* are structural protein genes that are necessary for muscle function. We chose to assay at 4 h post–heat shock when Etv2 expression is at a maximum (Figure S2). By ISH, *myod1* (Figure 2D), *myog*, and *myf6* (Figure S5) were almost extinguished by Etv2 overexpression, while *mylpfa* and *tnnt3c* were reduced but less so (Figure S5). qPCR demonstrated that all five of these muscle genes are significantly reduced by Etv2 expression (Figure 2E). When we examined these genes at later time points, expression had partially recovered, consistent with the fact that not all trunk cells go on to express vascular-specific genes (unpublished data). These data demonstrate that Etv2 is sufficient to both induce vascular genes and repress muscle genes, suggesting it may be sufficient to switch the fate of muscle cells into the vascular lineage.

Previously we reported that Fli1a and Tal1 could partially rescue angioblast development downstream of Etv2 [24] and both have been found to induce ectopic angioblasts in the early embryo using mRNA injection at the one-cell stage [25]–[27]. We wanted to test whether they were sufficient to induce ectopic *kdrl:GFP* in the trunk of late stage embryos similar to Etv2. To accomplish this we generated transgene constructs consisting of the *hsp70l* promoter driving expression of each transcription factor fused to *mCherry* and flanked by Tol2 transposable element sites. We injected these plasmids along with *tol2* mRNA into *kdrl:GFP* transgenic embryos to generate robust transient transgenic heat shock–inducible embryos. To test the activity of the Fli1a-mCherry and Tal1-mCherry fusion proteins, we heat shocked the embryos at shield stage and examined them at 16-somite stage for ectopic *kdrl:GFP* expression. Both were capable of inducing ectopic expression (Figure S6) as reported previously with RNA injection of untagged protein [25]–[27]. As a positive control we injected the *hsp70l:etv2* plasmid and demonstrated strong ectopic GFP expression similar to that seen in the germline transgenic (Figure S6). When heat shocked at 24 hpf, all three transgenes exhibited robust nuclear mCherry expression in the trunk 3 h post–heat shock (Figure S6). However, neither Fli1a nor Tal1 was able to induce ectopic *kdrl:GFP* expression (Figure S6). This finding highlights the unique ability of Etv2 to induce multiple endothelial genes and potently drive cells into this lineage at relatively late developmental stages.

### Fast Skeletal Muscle Transforms into Functional Endothelium Following Etv2 Expression

**Figure 3:**
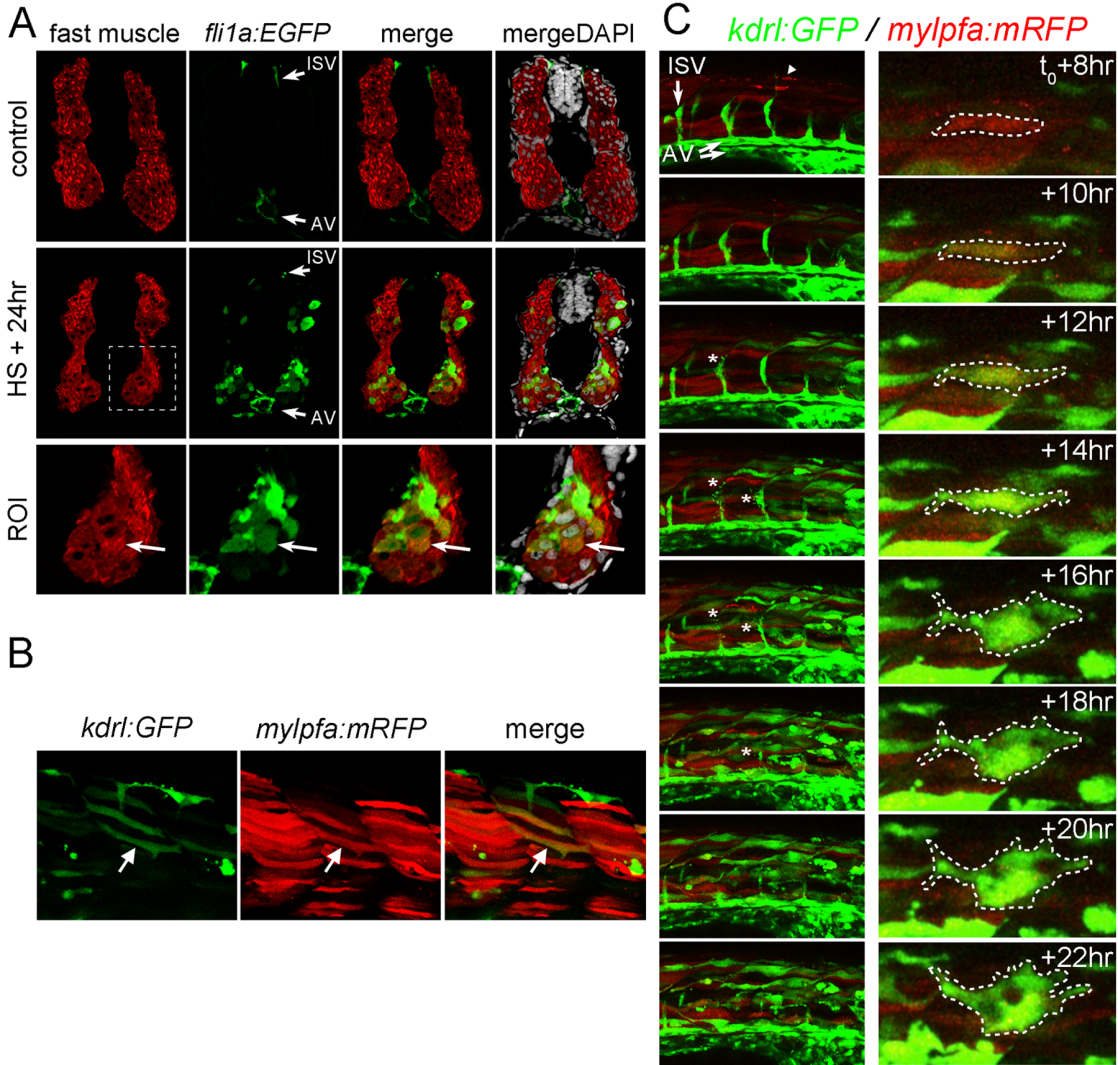
Fast skeletal muscle expresses ectopic endothelial genes following Etv2 overexpression. (A) Immunostained sections through the trunk of 48 hpf *hsp70l:etv2/fli1a:EGFP* embryos that were untreated (control) or heat shocked at 24 hpf (HS+24 h). Sections were stained for GFP and fast muscle myosin. Nuclei are stained with DAPI in the mergeDAPI panels. *fli1a:EGFP* is normally expressed in the intersomitic vessels (ISVs) and axial vessels (AVs) of control sections. However, following heat shock, many fast muscle myosin positive cells were also GFP positive (A). ROI is the region of interest highlighted by the dashed box in each panel. One section from 20 different embryos was observed for each treatment group with similar results within each group. (B) Confocal projection images of a *kdrl:GFP* ^+^ and *mylpfa:mRFP* ^+^ double positive muscle fiber (arrow) in a living embryo 12 h post–heat shock. (C) Time lapse imaging of the trunk (left column) and at the single cell level (right column) of a *mylpfa:mRFP/hsp70l:etv2/kdrl:GFP* triple transgenic embryo beginning at 8 h post–heat shock (t_0_+8 h). Heat shock occurred at 24 hpf. A few Etv2-mCherry^+^ nuclei are present in the first panel (arrowhead). The normal GFP^+^ intersomitic vessels (ISVs) and axial vessels (AVs) are labeled. *mylpfa:mRFP* labels fast muscle fibers in red. In the trunk, GFP expression first appears in muscle fibers between t_0_+8 h to t_0_+10 h and progresses in an caudal to rostral wave. mRFP^+^ fibers induce GFP expression and then soon switch off mRFP expression. ISV sprouts appear to apoptose and regress (asterisks). At the single cell level, mRFP^+^ fibers become GFP^+^ and then change morphology, a single cell is highlighted by a dashed outline in the right column.

We next wanted to identify the specific cell type that was responding to Etv2 overexpression. The shape of the induced *kdrl:GFP^+^* cells was reminiscent of muscle fibers (Figure 1). In the zebrafish embryo at this stage there are two well-defined muscle subtypes, termed fast and slow fibers. Slow fibers originate near the midline but migrate out to populate the outer layer of muscle adjacent to the skin. Fast fibers make up the bulk of the embryonic trunk musculature and are derived from the lateral paraxial mesoderm. Using muscle subtype-specific antibodies, we examined where ectopic GFP was expressed in 48 hpf *hsp70l:etv2/fli1a:EGFP* embryos that were heat shocked 24 h earlier (Figures 3A and S7; *kdrl:GFP* and *fli1a:EGFP* gave similar results). In Figure S7, slow muscle fibers are strongly labeled at the outer border of the myotomes, while fast muscle fibers located more medially are labeled more weakly. We never observed co-staining of GFP and slow muscle fibers in 20 sections from 20 separate embryos (Figure S7). Most of the ectopic GFP expression was present in the fast muscle population as labeled by a fast muscle myosin heavy chain-specific antibody in Figure 3A. All of the observed embryos exhibited GFP expression in fast muscles (*n* = 20). We also noted that some strongly GFP positive cells located within the fast muscle domain appeared to have reduced or no fast muscle myosin expression by 24 h post–heat shock suggesting these cells might be losing their muscle cell fate (Figure S8). Non–heat shocked controls never exhibited GFP expression within the muscle cell population (unpublished data).

If the fast muscle population was switching on expression of *kdrl:GFP*, we should be able to visualize this switch using time-lapse imaging of transgenic fish. We crossed *kdrl:GFP*/*hsp70l:etv2* fish with *mylpfa:mRFP* fish [28] that label fast muscle fibers and selected for embryos that expressed all three transgenes. We then imaged the trunk of these fish beginning at 8 h post–heat shock (t_0_+8 h) through 22 h post–heat shock (Figure 3C and Movie S2). Control embryos, both heat-shocked nontransgenic and non–heat shocked transgenic, display normal vascular development and strong mRFP expression in muscle with no GFP/mRFP overlap (unpublished data and Movie S1). In heat shock–treated embryos, we observed some residual nuclear Etv2-mCherry expression at t_0_+8 hr (Figure 3C, top left panel, arrowhead), but this expression was extinguished by t_0_+10 h. Over the course of the 22 h observation, many mRFP^+^ muscle fibers switch to GFP^+^ fibers (Figure 3C, left column). Occasionally, GFP^+^ fibers began to morphologically change and then appeared to undergo apoptosis. High-magnification observation of a single muscle fiber shows the mRFP^+^ cell becoming GFP^+^ and dramatically changing shape (Figure 3C, right column and Movie S3). Examination of single slices from the confocal imaging confirms that mRFP and GFP are co-expressed in some muscle fibers (Figures 3B and S8). These results support the immunostaining finding that fast muscle fibers are induced to express vascular genes following Etv2 expression.

Another interesting observation derived from time-lapse imaging is that normal angiogenic intersomitic sprouts appear to undergo apoptosis following Etv2 overexpression (Figure 3C, left column, asterisks). A high-resolution image of this event is presented in Figure S9. In fact, intersomitic vessels are largely absent in the region of *kdrl:GFP* ectopic expression, while the axial vessels are largely intact (see Figure 1A). This result may suggest that Etv2 must be tightly regulated for angiogenesis to occur or that angiogenic sprouts are especially sensitive to Etv2 overexpression.

Since the appearance of *kdrl:GFP* expression correlated with the disappearance of Etv2-mCherry, we tested whether continued expression of Etv2 blocked the expression of *kdrl*. We performed heat shocks every 12 h to maintain Etv2 expression and imaged the embryos at 72 hpf. Embryos receiving one or two heat shocks displayed abnormal vasculature and what appear to be transdifferentiated muscle fibers (Figure S9). However, embryos receiving four heat shocks, to maintain Etv2 expression, exhibit severe reductions in the normal expression of *kdrl:GFP* in the intersomitic vessels and none of the ectopic expression seen following a single heat shock (Figure S9). Control embryos heat shocked four times were similar to non–heat shocked control embryos (unpublished data). This suggests that a pulse of Etv2 expression is necessary for muscle fibers to express vascular genes and that maintained expression is either toxic to or reverts mature intersomitic vasculature to a non-*kdrl:GFP* positive state.

**Figure 4:**
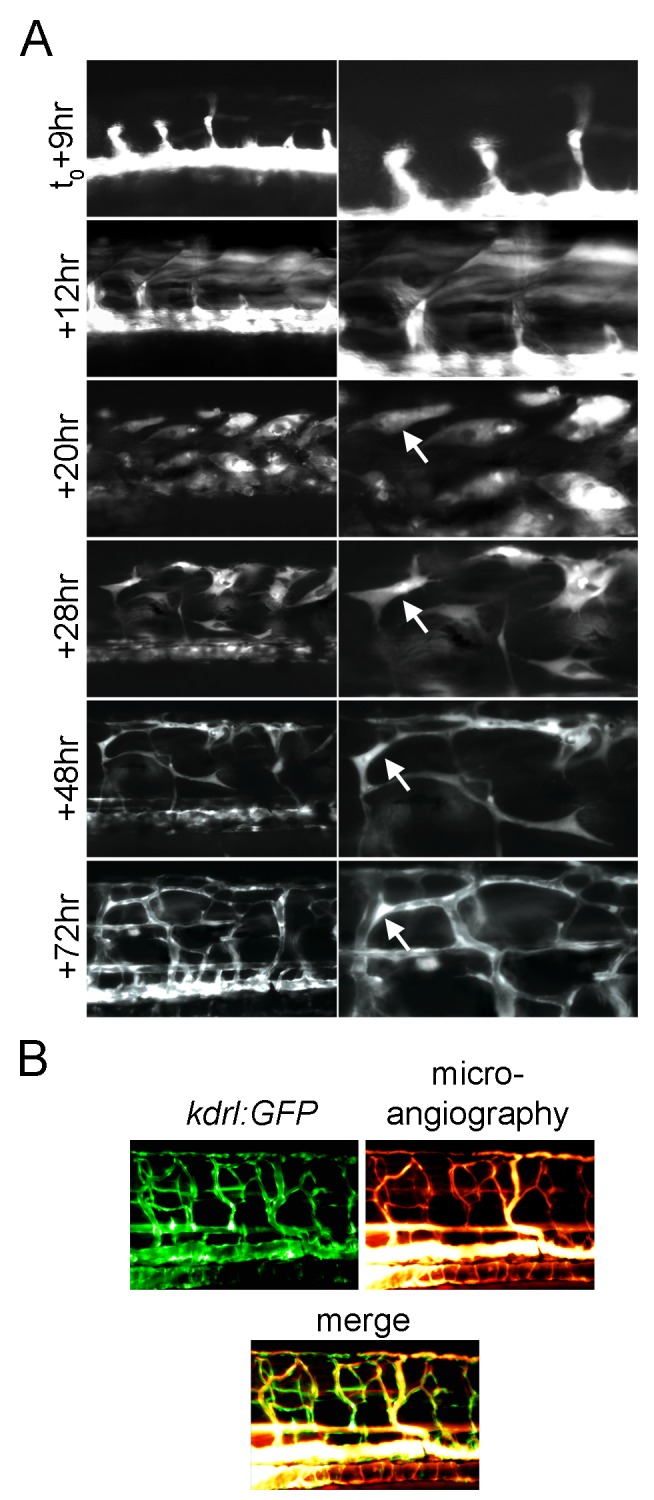
Fast skeletal muscle converts to functional endothelial cells following Etv2 expression. (A) Extended time lapse imaging of the trunk of a *kdrl:GFP/hsp70l:etv2* embryo showing ectopic GFP expressing cells change morphology from fiber-like (+12 h) to spindle-like (+20 h, +28 h) to form a network of thin cells (+48 h) and finally appear to form lumenized vessels (+72 h). The two panels shown at each time point are two magnifications of the same image. (B) Microangiography demonstrates the newly formed vessels are functional. Rhodamine dextran dye was injected into the circulation of a 4 dpf (+72 h post–heat shock) *kdrl:GFP/hsp70l:etv2* embryo. Rhodamine labels within all of the newly formed vessels and no vascular leakage are observed.

We next wanted to examine if these Etv2-induced cells were true angioblasts. Using long-term time-lapse microscopy, we observed the *kdrl:GFP^+^* cells over 3 d post–heat shock (Figure 4A). After the initial induction of GFP, the cells undergo major morphological changes. First the cells change from cuboidal fibers to “fat” spindle-shaped cells. Large vacuoles appear within these cells. They then extend long processes. The cells form an interconnected network of very thin cells with newly developed contacts to the remaining axial vessels by 2 d post–heat shock. And finally, by 3 d post–heat shock, the cells have lumenized to form an intact vascular network. To confirm that this new vasculature was functional we performed microangiography (Figure 4B). Fluorescent rhodamine tracer clearly perfused into even the thinnest of the newly formed *kdrl:GFP^+^* vessels.

Although our imaging data clearly suggested that fast muscle fibers were the cellular source for these newly derived blood vessels, we wanted to eliminate the possibility that angiogenic sprouts from unseen surviving vasculature resulted in the observed new vasculature. Therefore we performed lineage tracing of fast muscle fibers in Etv2 overexpressing embryos. To achieve this we crossed *kdrl:GFP/hsp70l:etv2* fish to *ubi:Switch* fish and injected them with plasmid encoding fast muscle–specific *mylpfa:cre-ERt2*. *Ubi:Switch* fish contain a floxed *GFP* cassette upstream of *mCherry* driven by the ubiquitous *ubi* promoter [29]. Expression of Cre recombinase switches cells containing this transgene from green (GFP^+^) to red (mCherry^+^). Hydroxytamoxifen (5 µM) was added immediately after heat shock to induce Cre activity. Injection of the *mylpfa:cre-ERt2* plasmid into *ubi:Switch* fish resulted in many muscle fibers switching on mCherry expression with little to no nonmuscle switching (unpublished data). In heat-shocked *kdrl:GFP*/*hsp70l:etv2*/*ubi:Switch* embryos, many mCherry^+^ muscle fibers were observed and 5/15 embryos exhibited *kdrl:GFP^+^*, mCherry^+^ blood vessels (Figure S10). Embryos that were not heat treated did not display mCherry^+^ blood vessels (0/13) but had robust switching in muscle fibers (Figure S10).

**Figure 5:**
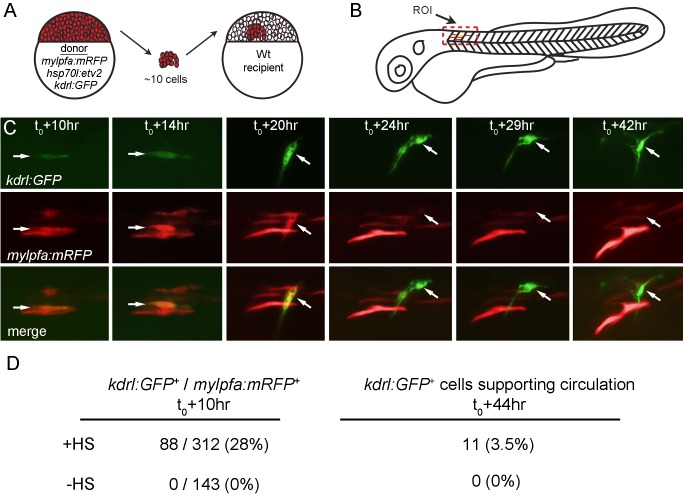
Etv2 cell autonomously initiates transdifferentiation of muscle cells. (A) Blastula cell transplantation was performed from triple transgenic, *mylpfa:mRFP/hsp70l:etv2/kdrl:GFP^+^*, into wild-type embryos. Approximately 10 cells were transplanted per embryo. Transplanted embryos were raised until 22 hpf, at which point they were selected for embryos displaying *mylpfa:mRFP* expression in distinct regions absent in *kdrl:GFP*, region of interest (ROI) boxed in (B) corresponds to images in (C). These embryos were then either heat shocked or left as no heat shock controls. Embryos were then analyzed for *mylpfa:mRFP/kdrl:GFP* coexpression at 10 h post–heat shock and followed out to 42 h post–heat shock (C). A muscle cell labeled with the arrow undergoes transdifferentiation to form a lumenized vessel (C). (D) Quantification of transdifferentiation efficiency per muscle cell. Only clearly distinguishable muscle cells were counted. Thirty-eight chimeric embryos, 312 total cells, were observed in the heat-shocked condition, and 20 chimeric embryos, 143 total cells, were observed for the control non–heat shocked condition.

To demonstrate the muscle to endothelial switch more clearly and estimate the efficiency of transdifferentiation, we used blastula transplantation to create chimeric animals with small numbers of transgenic cells within a wild-type background (Figure 5A and B). Approximately 10 *Kdrl:GFP/hsp70l:etv2/mylpfa:mRFP* ^+^ cells were transplanted into each wild-type embryo. These embryos were then raised and either heat shocked at 22 hpf or left as non–heat shocked controls. Only embryos that exhibited clearly defined *mylpfa:mRFP* expression in distinct cells with no surrounding *kdrl:GFP* expression at the time of heat shock were analyzed (heat shock *n* = 38, non–heat shock *n* = 20). By 10 h post–heat shock, *kdrl:GFP^+^/mylpfa:mRFP^+^* cells can be observed although the *mylpfa:mRFP* is weak due to the rapid decrease in expression following Etv2 expression (Figures 5C and S11). Around 20 h post–heat shock, some of the *kdrl:GFP^+^* cells start taking on a branched morphology and by ∼40 h these cells take on a lumenized morphlolgy (Figures 5C and S11). In fact, these cells have successfully integrated into the existing vascular network and support flow of blood cells (Movie S4). We calculated that 28% (88/312 cells) of *mylpfa:mRFP^+^* muscle fibers induced *kdrl:GFP* and 12.5% (11/88 cells) of these cells became part of functional vessels or 3.5% of the total muscle fibers observed (11/312 cells) (Figure 5D). Cells that induced *kdrl:GFP* but that did not change morphological shape seemed to fade out over time or disappear, suggesting that they either switch back to the muscle fate or die. *Kdrl:GFP^+^* never appeared in the *mylpfa:mRFP^+^* population of non–heat shocked embryos (0/143 cells) (Figure 5D). These results convincingly demonstrate that Etv2 expression can cell autonomously induce fast skeletal muscle to transdifferentiate into functional vasculature in the trunk of zebrafish embryos.

### VEGF Signaling Is Dispensable for Induction but Modulates the Morphology of Newly Induced Angioblasts

**Figure 6:**
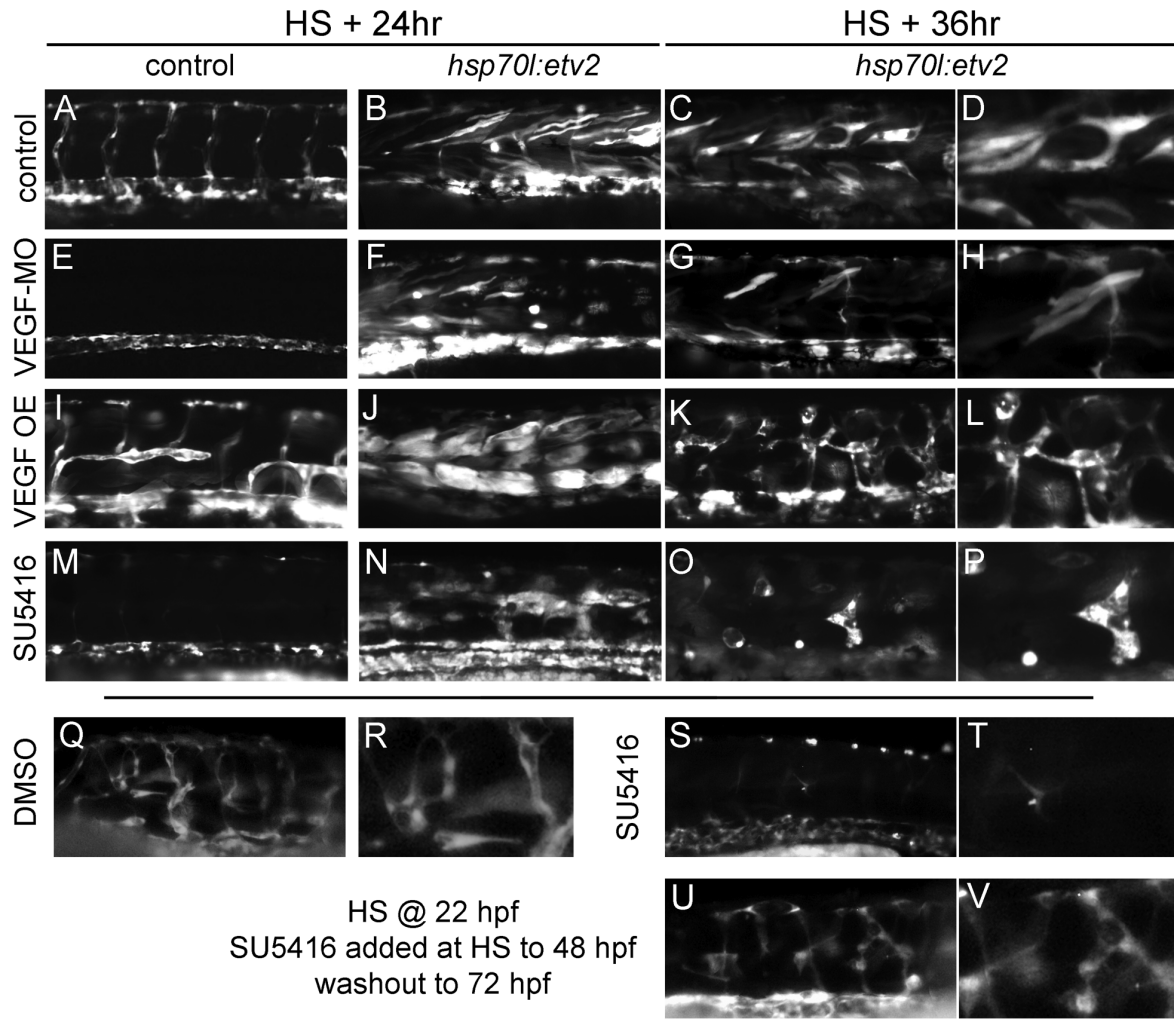
VEGF signaling is dispensable for induction but necessary for maturation of Etv2 induced vasculature. (A–D) Control *kdrl:GFP* embryos (A) or *kdrl:GFP/hsp70l:etv2* (B–D) embryos following heat shock at 24 hpf and imaged at 24 h or 36 h post–heat shock. Control embryos exhibit normal vascular *kdrl:GFP* expression (A), while *kdrl:GFP/hsp70l:etv2* embryos exhibit the ectopic GFP and morphological changes previously described (B–D). (E–H) VEGFAa morpholino (VEGF-MO) treated embryos lack intersomitic vessels (E) but still induce *kdrl:GFP* in muscle fibers (F). However, *kdrl:GFP* ^+^ muscle fibers do not undergo the normally observed morphological changes following heat shock–induced expression of Etv2 (G,H). (I–L) Overexpression of VEGFAa^121^ (VEGF OE) driven by the *hsp70l* promoter results in disorganization and expansion of the normal vasculature (I). Following heat shock–induced expression of Etv2, no significant change in the number of muscle fibers expressing *kdrl:GFP* is observed (J). However, the morphological changes observed are accelerated in the presence of elevated VEGF (K,L). (M–P) Treatment of embryos with SU5416, a Kdr inhibitor, similarly inhibits intersomitic vessel development in control embryos (M). However, drug treatment does not inhibit induction of *kdrl:GFP* following heat shock–induced Etv2 expression in muscle (N). The morphology and survival of these fibers is compromised when Kdr is inhibited (O,P). (Q–V) Removal of VEGF inhibitor SU5416 24 h following heat shock allows for survival and maturation of transdifferentiated cells. *Kdrl:GFP/hsp07l:etv2* embryos were heat shocked at 22 hpf and then treated with DMSO carrier or SU5416 for 24 h at which point the drug was either maintained (S,T) or removed (U,V) and the embryos were allowed to develop until 72 hpf. (Q,R) DMSO controls exhibit a transdifferentiated vascular network similar to that in untreated controls. (S,T) Sustained SU5416 treatment largely abolishes the *kdrl:GFP^+^* vascular network. (U,V) Removal of SU5416 24 h post–heat shock results in the development of a vascular network similar to controls (Q,R), suggesting VEGF signaling modulates the survival and maturation of muscle-derived vessels and not the initial induction. For all experiments at least 20 embryos were observed with similar results.

Since *kdrl* and *kdr*, VEGF receptors, are induced following Etv2 overexpression in muscle (Figure 2) and the VEGFA ligand has been previously reported to be expressed in muscle during the competency window we defined for transdifferentiation [22], we further investigated if VEGF signaling is necessary for this process. First, we asked if inhibition of VEGF signaling has an effect on the induction of *kdrl:GFP* when Etv2 is overexpressed. Inhibition was accomplished in two ways: morpholino-mediated antisense knockdown of the VEGFAa ligand (VEGF-MO) and chemical inhibition of the VEGF receptors with SU5416. Treatment of control embryos with VEGF-MO resulted in lack of intersomitic vessels as reported previously (Figure 6E compared to Figure 6A) [30]. However, overexpression of Etv2 still resulted in *kdrl:GFP^+^* cells in the trunk, suggesting that VEGF signaling is not necessary for induction of transdifferentiation (Figure 6F compared to Figure 6B). Though with later observations (36 h post–heat shock), we found that Etv2-induced *kdrl:GFP^+^* cells failed to undergo the morphological changes seen in control embryos when VEGF-MO was added (Figure 6G,H compared to Figure 6C,D). To test this finding in another way, we used SU5416 to inhibit Kdr, the VEGF receptor [31]. Similar to VEGF-MO treatment, chemical inhibition of VEGF signaling did not prevent *kdrl:GFP* expression following overexpression of Etv2 (Figure 6N versus Figure 6B), but was able to limit or delay intersomitic vessel sprouting as expected in control embryos (Figure 6M versus Figure 6A). However, 36 h post–heat shock many of the Etv2-induced *kdrl:GFP^+^* cells had disappeared and those that remained were not similar in morphology to either control or VEGF-MO–treated embryos (Figure 6O,P versus Figure 6C,D,G,H). This difference between VEGF-MO treatment and chemical inhibition may be the result of more efficient signal blockage by SU5416 since we only targeted one of several VEGF ligands with the morpholino while the inhibitor should block signaling from both Kdr and Kdrl in zebrafish. These results suggest that VEGF signaling is not necessary for induction but plays a later role in the survival and morphological changes associated with Etv2 overexpression. To further test this, we specifically inhibited VEGF signaling during the first 24 h post–heat shock using SU5416 and then washed out the inhibitor and allowed the embryos to develop until 72 hpf. Embryos treated with DMSO exhibit transdifferentiation similar to untreated controls (Figure 6Q,R). Embryos treated with SU5416 from heat shock at 22 hpf until 72 hpf have almost no *kdrl:GFP^+^* vasculature in the trunk (Figure 6S,T), although they initially induce these cells (Figure 6N). When SU5416 is washed out 24 h post–heat shock, the survival of the muscle-derived vasculature is rescued and they form an interconnected network similar to DMSO controls (compare Figure 6U,V with Figure 6Q,R). This result supports the conclusion that VEGF is necessary for the survival and maturation of muscle-derived endothelial cells but not for their induction.

We also wanted to explore whether increased VEGF signaling could enhance transdifferentiation of muscle into endothelium. To activate VEGF signaling, we chose to overexpress the VEGFAa^121^ [32] diffusible ligand using a transient transgenic approach (VEGF OE). We again used the *hsp70l* promoter, this time to drive expression of the VEGF ligand following heat shock. Overexpression of VEGFAa^121^ itself resulted in aberrant angiogenesis of intersomitic vessels but never ectopic *kdrl:GFP* expression in muscle fibers (Figure 6I). VEGF OE coupled with Etv2 overexpression resulted in robust *kdrl:GFP* expression 24 h post–heat shock (Figure 6J) and accelerated morphological changes at 36 h post–heat shock (Figure 6K,L). The morphological changes seen at 36 h with VEGF OE were reminiscent of those seen at 48 h post–heat shock with Etv2 only. However, heat shock induction of both Etv2 and VEGFAa^121^ together at time points later than 30 hpf did not result in ectopic expression of *kdrl:GFP* in muscle, suggesting again that VEGF signaling does not extend the competency of muscle cells to respond to Etv2 (unpublished data).

From these results we conclude that VEGF signaling is not necessary for the induction of transdifferentiation by Etv2 overexpression. However VEGF signaling is necessary for the maturation of these induced cells into endothellial cell-lined vascular networks. Also, activation of VEGF signaling can accelerate the maturation of these induced cells but does not expand the competency for transdifferentiation.

### Wnt Signaling Is Necessary for Etv2-Induced Transdifferentiation

We next wanted to know if any common developmental signaling pathways were necessary for Etv2-induced transdifferentiation. Given the zebrafish models utility in drug screens, we decided to test commonly used pathway inhibitors for either increased or decreased *kdrl:GFP* expression following Etv2 overexpression. We tested multiple pathways including the BMP, Notch, and Wnt pathways described to be upstream of Etv2 in embryonic stem cell cultures [5]. Wnt modulators had the most robust effects in our in vivo transdifferentiation model and were further studied.

**Figure 7:**
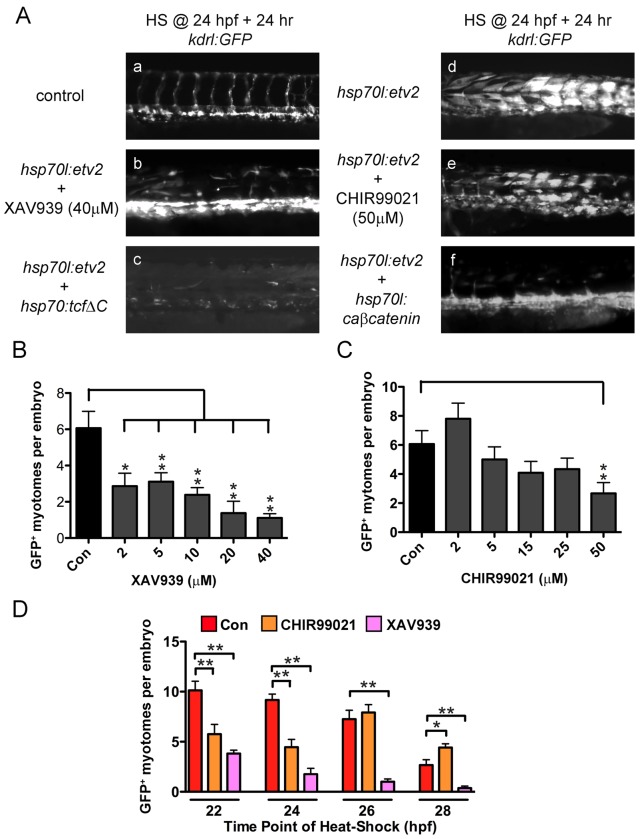
Canonical Wnt signaling is necessary for Etv2-induced transdifferentiation of muscle fibers. (A) Inhibition of Wnt signaling using XAV939 or heat shock–inducible, dominant negative Tcf3 (*hsp70l:tcfΔC-EGFP*) blocks Etv2-induced *kdrl:GFP* expression. However, activation of Wnt signaling using CHIR99021 or heat shock–inducible, constitutively active β-catenin (*hsp70l:caβ-catenin-2A-TFP*) also blocks Etv2-induced *kdrl:GFP* expression. (B) XAV939 dose dependently inhibits Etv2-induced *kdrl:GFP* expression. *Kdrl:GFP* expression was quantified by counting the number of GFP^+^ myotomes with a myotome being considered positive if >3 muscle fibers within a given myotome were GFP^+^. (C) CHIR99021 dose dependently inhibits Etv2-induced *kdrl:GFP* expression. (D) XAV939 (40 µM) inhibits Etv2-induced *kdrl:GFP* expression independent of heat shock time, while CHIR99021 (50 µM) inhibits at 22 and 24 h heat shocks, has no effect at 26 h heat shock, but enhances Etv2-induced *kdrl:GFP* expression at 28 h heat shock. For all data *t* test was used for statistical comparisons with (*) *p*<0.05 and (**) *p*<0.01.

To examine the interaction of the Wnt pathway with Etv2 overexpression in detail, we utilized both pharmacologic and genetic tools. First, we treated *hsp70l:etv2/kdrl:GFP* fish with XAV939, a chemical inhibitor of the Wnt pathway that acts by indirectly stabilizing the β-catenin degradation complex by inhibiting tankyrase enzymes that activate the degradation of Axin [33]. Treatment of heat-shocked embryos with XAV939 significantly decreased the number of myotomes that responded to Etv2 overexpression by expressing *kdrl:GFP* (Figure 7Ab compared to 7Ad). Doses of XAV939 between 2 and 40 µM significantly inhibited kdrl:GFP expression in response to Etv2 expression (Figure 7B). A genetic method to inhibit canonical Wnt signaling is through the overexpression of a dominant negative Tcf. We used a heat shock–inducible line, *hsp70l:tcfΔC-EGFP* [34]. Crossing *hsp70l:tcfΔC-EGFP* into the *hsp70l:etv2/kdrl:GFP* fish resulted in a near complete inhibition of ectopic GFP expression following heat shock (Figure 7Ac compared to 7Ad; the TcfΔC-EGFP is degraded by this stage). Since Wnt inhibition blocked Etv2 responsiveness, we reasoned that activation of the pathway should enhance responsiveness. To test this we used the Wnt activator CHIR99021 [35]. This chemical acts by inhibiting GSK3β phosphorylation of β-catenin resulting in its stabilization. Stabilized β-catenin is then able to move to the nucleus and activate its target genes. Surprisingly, addition of CHIR99021 to heat-shocked embryos dose dependently inhibited *kdrl:GFP* expression in muscle fibers (Figure 7Ae compared to 7Ad; quantified in Figure 7C). To test this confusing result in a different way, we turned to a new heat shock–inducible transgenic line that expresses a phosphorylation and degradation resistant constitutively active β-catenin (*hsp70l:caβ-catenin-2A-TFP*) to activate Wnt signaling. Following heat shock, caβ-catenin suppressed *kdrl:GFP* expression in muscle fibers nearly as well as TcfΔC and more completely than CHIR99021 (Figure 7Af). These results were surprising since both Wnt inhibition and Wnt activation blocked transdifferentiation.

**Figure 8:**
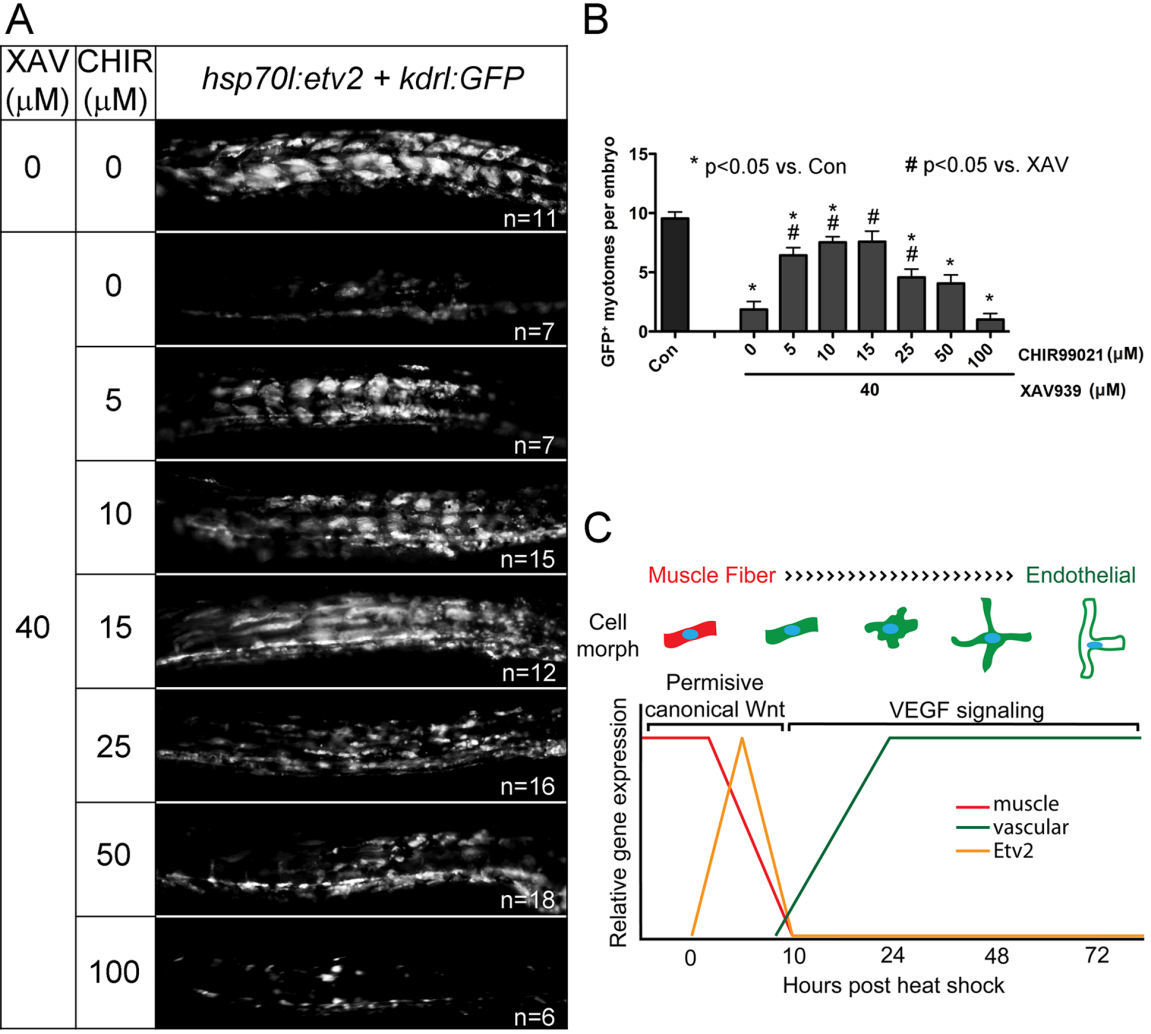
A tightly controlled level of Wnt signaling is necessary for muscle to transdifferentiate into endothelial cells. (A and B) Dose-dependent rescue of XAV939 inhibition of *kdrl:GFP* expression by the addition of Wnt activator CHIR99021. (A) Images of the trunk of 48 hpf embryos following treatment with the noted compounds and heat shock at 24 hpf. Note that 40 µM XAV939 alone almost completely inhibits ectopic GFP expression and the addition of 5–25 µM of CHIR99021 can rescue this inhibition with 15 µM being the most affective dose. CHIR99021 doses greater than 50 µM do not rescue XAV939 inhibition. (B) Quantification of the observations in (A). *t* test, (*) *p*<0.05 comparing Control (Con) to drug treated and (#) *p*<0.05 comparing XAV939 (40 µM) to XAV939 (40 µM) plus varying doses of CHIR99021. (C) Schematic of muscle cell transdifferentiation into endothelial cells. Muscle cells exhibiting permissive levels of canonical Wnt activity are responsive to a pulse of Etv2 expression, resulting in repression of muscle gene expression and initiation of vascular gene expression. Cells expressing vascular genes respond to VEGF signals and change morphology from muscle fiber to thin multibranched and finally to lumenized patent endothelium.

Since the response of muscle cells to Etv2 expression changed over time, we reasoned that the cells might also change in responsiveness to Wnt modulation over time. We treated embryos with XAV939 or CHIR99021 and heat shocked them at 22, 24, 26, and 28 hpf. XAV939 significantly inhibited *kdrl:GFP* expression at all time points (Figure 7D). However, CHIR99021, which repressed *kdrl:GFP* expression when heat shock was administered at 22 hpf, actually increased *kdrl:GFP* expression when treated at 28 hpf (Figure 7D). This result suggested that Wnt signaling needed to be active but not over a certain threshold for transdifferentiation to occur. Recently reported Wnt signaling reporter transgenic zebrafish have GFP expression in an anterior to posterior, low to high, gradient in the somites at 24 hpf with expression resolving to the tip of the tail over the next 24 h [36]. This suggested to us that Wnt signaling at 24 hpf is near the upper limit of the Wnt activity threshold for transdifferentiation. To examine this hypothesis in more detail, we used pharmacological manipulation of the Wnt pathway to dissect the range of Wnt activity necessary for transdifferentiation when heat shock was applied at 24 hpf. Addition of XAV939 (40 µM) inhibited Wnt signaling and resulted in a near complete loss of Etv2 responsiveness (Figure 8A). CHIR99021 was then added in increasing doses to re-establish Wnt signaling. Between 5 and 25 µM CHIR99021 was able to significantly increase the response to Etv2 when co-treated with XAV939 (Figure 8A,B). A dose of 15 µM was able to rescue response back to levels seen in control embryos (Figure 8B). Doses of CHIR99021 greater than 50 µM were no different from treatment with XAV939 alone (Figure 8B). Additionally, we treated embryos heat shocked at 28 hpf, when their transdifferentiation response is minimal, with different doses of CHIR99021 to determine if an optimal activating Wnt signal could be found that would increase transdifferentiation at this time. Although CHIR99021 was able to expand the number of responding myotomes from ∼2 to ∼4 at doses similar to those used in Figure 7, increasing Wnt signaling did not improve the responsiveness of anterior myotomes (Figure S12), suggesting that the loss of responsiveness to Etv2 expression is independent of Wnt signaling. We also tested if Wnt activation could extend the temporal window of competency for muscle cells to respond to Etv2. However, treatment of *hsp70l:etv2/kdrl:gfp* embryos with different doses of CHIR99021 or using constitutively active β-catenin after 30 hpf never resulted in transdifferentiation of muscle fibers (unpublished data). These results demonstrate that a tightly controlled level and timing of Wnt signaling is necessary for fast muscle fibers to transdifferentiate into endothelial cells.

In summary, overexpression of Etv2 can induce fast muscle cells to transdifferentiate into functional endothelial cells in vivo. This competency is developmentally restricted to a time between 22 and 30 hpf and is dependent upon Wnt signaling. Following Wnt-dependent induction, VEGF signaling mediates the maturation and survival of transdifferentiating cells. Following 30 hpf, muscle cells become refractory to Etv2-induced transdifferentiation through an unknown mechanism. Our results are summarized in Figure 8C.

### Etv2 Induces Vascular Marker Gene Expression in C2C12 Cells

To determine if mammalian cells have a similar capacity for transdifferentiation, it was tested whether overexpression of Etv2 in the C2C12 mouse myoblast cell line could induce expression of vascular genes. As shown in Figure S13, similar to the zebrafish Etsrp/Etv2 overexpression, Etv2 could induce expression of Kdr, Fli1, Tal1, and VEcad in C2C12 cells. This ability of C2C12 cells to express vascular endothelial genes appears to be specific since three other tested cell types did not show the same capacity (Figure S13) and previous reports of limited Etv2 responsiveness in vitro [37] and in vivo [38]. These data suggest that Etv2 can be potentially used to transdifferentiate mammalian muscle cells into vascular cells.

## Discussion

Here we have described the ability of a single transcription factor, Etv2, to transdifferentiate fast skeletal muscle cells into functional vascular endothelium in vivo. The ability of differentiated muscle cells to become endothelium has not been described before. However, there is a close developmental relationship between these two cell types. In the zebrafish tail, a cell fate choice is made between somite and endothelium and this process is Wnt signaling dependent [34]. In chick and quail, it has been reported that angioblasts derived from the somites populate the endothelium of the dorsal aorta [39]. Although this relationship has not been demonstrated in other organisms, there have been reports of somite-derived angioblasts populating the limb vasculature in quail-chick chimeras [40] and mice [41]. All of these events occur at presomitic or early somitic stages and do not represent “transdifferentiation” per se, but are indicative of alternative fate choices during development. We propose we are observing true transdifferentiation since at the times we observed activation of vascular gene expression muscle cells have distinctive myotome spanning morphology, express mature muscle myosins, and are contractile.

The developmental window in which we observed this transdifferentiation, 22 hpf to 30 hpf, is quite late in myotome development. Although muscle cells are contractile and expressing muscle-specific genes in this window, they are still undergoing myofusion to become multinucleated and innervation from motor neurons is still expanding within the myotome. Could either of these process be the signal that terminates the competency window for transdifferentiation? This question awaits further study. Interestingly, we observed a wave of competency for transdifferentiation from anterior to posterior. This makes sense as somite development and myogenesis in the trunk normally progress in an anterior to posterior wave. However, wave-like progressions of morphological or signaling events at this late stage of myotome development are not well described. It will be very desirable to identify the factors that define the capacity of fast muscle cells to respond to Etv2.

Etv2 expression is dynamically regulated during development with peak expression when blood and vasculature is being specified [1],[5],[42]. We found that a pulse of Etv2 expression, but not maintained expression, was able to induce transdifferentiation of muscle cells. This suggests that Etv2 is a specifying factor that initiates a cascade of events driving endothelial development. Maintained Etv2 expression prevented transdifferentiation. In mouse embryos expressing Etv2 under the control of the *Mesp1* promoter in somites, no fate switch to endothelium occurred [38]. This may be due to persistent expression of Etv2. Alternately, the expression level in the *Mesp1:Etv2* mice might have been too low to induce fate switching. In either case, if Etv2 is to be used in regenerative medicine applications, its expression will need to be tightly regulated.

Using a targeted small molecule screen, we found that both the VEGF and Wnt signaling pathways were important for muscle cells to become functional endothelial cells. VEGF is a well-established mediator of endothelial cell morphogenesis and survival. We found that VEGF signaling is dispensable for induction of transdifferentiation but mediates the maturation and survival of Etv2-induced muscle cells. Importantly, transcripts encoding the VEGF receptor, Kdrl, did not appear until Etv2-mCherry expression was extinguished in the muscle fibers 8–10 h post–heat shock. This most likely corresponds with the onset of VEGF dependency.

Canonical Wnt signaling plays important roles in mesoderm specification and the specification of multiple cell types derived from this tissue [43]. In vascular development, Wnt signaling is critical for proper patterning and angiogenesis, but appears to be dispensable for angioblast specification [44]. This is in contrast to our findings in the muscle cell where Wnt signaling is critical for transdifferentiation. A possible answer to this conflict is that Wnt signaling may maintain muscle cells in a partially differentiated developmental state and Etv2 can act on this permissive substrate to specify angioblast. The Wnt signal may enable the cells to revert to a primitive mesodermal state. We found that Wnt signaling overactivation inhibited transdifferentiation just as efficiently as Wnt inhibition. Using a downstream agonist we could rescue transdifferentiation from an upstream antagonist within a defined range of doses. This highlights the sensitivity of cells to Wnt pathway modulation and suggests experiments using constitutively active or dominant negative constructs should be revisited with methods that can more sensitively regulate Wnt activity.

The point of interaction of Wnt signaling with Etv2 is currently unknown. The function of Wnt signaling in mesoderm development is complicated. Activation or repression of signaling at different developmental stages can result in opposite effects in the same cell population. In mouse ES cells, overexpression of Etv2 can increase the yield of angioblasts and hematopoietic cells [5]. This correlates with reduced Wnt signaling and addition of constitutively active β-catenin could respecify these cells back to the cardiac lineage [8], suggesting that Wnt signaling acts downstream of Etv2. In our experiments, Wnt signaling was altered at the time of heat shock with chemicals or in sync with heat shock using transgenic fish. The first measureable effects were seen with the induction of, or lack of induction of, *kdrl:GFP* at 8–10 h post–heat shock. This defines a small time window from which we can dissect this interaction in the future. Since activation of Wnt signaling alone is not sufficient for transdifferentiation, the Wnt signal must be in parallel or downstream of Etv2. In ES cells overexpression of Etv2 resulted in down-regulation of Wnt-related genes, but no direct interactions were established [8]. Future studies should focus on direct and indirect interactions between Etv2 and Wnt signaling and the dynamics of these interactions as they pertain to fate specification.

A recent report by Ginsberg et al. [37] demonstrated that Etv2 could reprogram human amniotic cells to an endothelial cell lineage and that these cells could functionally integrate into a mouse liver injury model. Overall our results suggest that myoblast cells may be a viable source for the derivation of endothelial cells, as supported by preliminary evidence that C2C12 is capable of being induced to express vascular marker gene expression but not other tested cells. It will be interesting to determine if primary mammalian myoblast cells can be transdifferentiated by transient expression of Etv2 into functional endothelial cells that support circulation. If so, it may be possible to use primary myoblasts as a source of cells for the treatment of vascular diseases.

## Materials and Methods

### Transgenic Zebrafish and Heat Shock

All transgenes were generated using the Tol2kit [45] and Multisite Gateway System (Life Technologies). To generate the *hsp70l:etv2* transgene, the zebrafish *etv2* open reading frame minus the stop codon was cloned into pDONR221 using BP clonase to generate pME-*etv2*NS. A LR clonase II Plus reaction was then performed with p5E-*hsp70l*, pME-*etv2*NS, p3E-*mCherry*pA, and pDESTTol2pA to generate the *Tg(hsp70l:etv2-mCherrypApAT2)* plasmid. This plasmid was then injected along with *tol2* mRNA (20 pg plasmid and 12 pg mRNA per embryo) into *kdrl:GFP* ^+/+^ embryos to generate *kdrl:GFP/hsp70l:etv2* founder fish. The offspring of injected fish were screened by heat shock for the expression of nuclear mCherry. Three out of 23 screened fish were positive for mCherry expression following heat shock, and of those three, we chose to maintain the line with the strongest expression and the ability to robustly induce *kdrl:GFP* expression following heat shock at dome stage (see Figure S1). The *Tg(hsp70l:fli1a-mCherry)*, *Tg(hsp70l:tal1-mCherry)*, and *Tg(hsp70l:VEGFAa^121^)* transgenes were generated using the method above and pME-*fli1a*NS, pME-*tal1*NS, or pME-*VEGFAa^121^* as the middle entry. p3E-polyA was used for *Tg(hsp70l:VEGFAa^121^)* instead of the p3E-mCherrypA. Full-length zebrafish *fli1a* and *tal1*, the longer alpha isoform, were used to generate the middle entry vectors without stop codons. The *Tg(mylpfa:cre-ERt2)* transgene was generated using p5E-*mylpfa*, pME-*cre-ERt2*, p3E-*pA*, and pDESTpAT2. Hydroxytamoxifen (5 µM) was added immediately after heat shock to the fish water to induce recombination in lineage tracing experiments. The *Tg(hsp70l:caβ-catenin-2A-TFP)* line was produced using *Xenopus laevis* β-catenin with mutations in the GSK3 phosphoryation site that renders it constitutively active [46], and fusing it in frame to the 2A peptide [47] and TFP. This protein coding sequence was placed behind the *hsp70l* promoter in a Tol2 vector. *Et(tal1:GFP)* was isolated by us in an enhancer trap screen (unpublished data). *Tg(mylpfa:mRFP)* was generated by cloning the *mylpfa* promoter sequence from genomic DNA into the Tol2mini vector [48] harboring the mRFP sequence. Established zebrafish lines used were *Tg(kdrl:GFP)^la116^* [49], *Tg(fli1a:EGFP)^y1^* [23], *Tg(ubi:Switch)* [29], and *Tg(hsp70l:tcfΔC-EGFP)^w74^* [34]. All zebrafish use was approved by the University of California, Los Angeles Animal Care and Use Committee.

Heat shock was performed at 38.5°C by placing the zebrafish embryos in a 3 cm plastic dish with 2 mL of water, sealing the dish with parafilm, and floating it upon the surface of a 38.5°C water bath for 30 min. The dish was then placed at 28.5°C until observations were taken.

### Drug and Morpholino Treatments

SU5416, CHIR99021, and XAV939 were obtained from Sigma and diluted in DMSO. DMSO concentration in control or experimental conditions did not exceed 0.7%. VEGFAa or standard control morpholino was used at 8 ng per embryo as previously described [30]. VEGF-MO, 5′-GTATCAAATAAACAACCAAGTTCAT-3′; control-MO, 5′-CCTCTTACCTCAGTTACAATTTATA-3′.

### Immunostaining and in Situ Hybridization

Immunostaining was performed on 8 µm cryosections as previously described [50]. Anti–slow muscle myosin, clone f59, and anti–fast muscle myosin, clone MF20, were used at 1∶50 dilution and obtained from the Developmental Studies Hybridoma Bank. Anti-GFP (A6455), 1∶500 dilution, anti-rabbit Alexa488 (A11070), 1∶200 dilution, and anti-mouse Alexa555 (A21422), 1∶200 dilution, were obtained from Life Technologies. Sections were mounted using Prolong Gold with DAPI (Life Technologies). In situ hybridization was performed as previously described [51].

### qRT-PCR

Real-time qPCR was performed using FastStart SYBR Green Master Mix (Roche) on a Stratagene Mx3005P qPCR system. RNA was isolated using Trizol reagent (Invitrogen) and cDNA generated using Superscript III reverse transcriptase (Invitrogen) with oligo dT primers. Gene expression levels were calculated relative to uninjected controls as previously described [52]. Three independent biological samples were analyzed in duplicate for each experimental and control group. Student's *t* test was used to determine significance with *p*<0.05. Gene-specific primers are listed in Table S1.

### Cell Transplantation

Transplantation of *kdrl:GFP/hsp70l:etv2/mylpfa:mRFP* cells into wild-type embryos was performed as described by Kemp et al. [53].

### Microscopy and Imaging

Images were captured on an Axioskop 2 plus microscope (Zeiss) or a Stemi2000-C (Zeiss) using 5× or 10× objectives with an AxioCam camera and Openlab 4.0 software (Improvision). Confocal images were collected on a Zeiss LSM 510 with a 20× water objective. Adobe Photoshop was used to adjust brightness and contrast and assemble composite images.

## Supporting Information

Figure S1 *Hsp70l:etv2* is as effective as *etv2* mRNA injection at inducing *kdrl:GFP* expression. (A) A shield stage embryo that was heat shocked at dome stage and imaged for mCherry expression. Nuclear-localized Etv2-mCherry is visible. (B) Heat shock of *hsp70l:etv2-mCherry* transgenic embryos at dome stage induces *kdrl:GFP* expression at tailbud stage similar to that seen with mRNA injection. (C) mRNA injection of *etv2* induces *kdrl:GFP* expression at tailbud stage. The embryos overexpressing Etv2 in (C) and (D) gastrulated abnormally due to overexpressed Etv2.
(TIF)

Figure S2 qRT-PCR quantification of Etv2-mCherry following heat shock. Etv2-mCherry expression is very high 4 h post–heat shock but quickly decreases to levels similar to control by 24 h post–heat shock. This time course of expression is very similar to that observed by nuclear Etv2-mCherry fluorescence.
(TIF)

Figure S3 *Kdrl:GFP*, *fli1a:EGFP* and *tal1:GFP* are all induced in the trunk of hsp70l:etv2 embryos following heat shock. *Hsp70l:etv2* was crossed into *kdrl:GFP* (A, B), *fli1a:EGFP* (C, D), or *tal1:GFP* (E, F), and the resulting embryos were either left at control temperature or were heat shocked at 22 hpf and then imaged at 48 hpf. Control embryos never exhibited ectopic GFP expression in any group. Heat-shocked embryos (+HS) always exhibited ectopic GFP expression in the trunk (right column is high magnification image of the trunk corresponding to the adjacent embryo in the left column), although *tal1:GFP* was significantly weaker than the other two transgenes. At least 20 embryos were observed for each treatment with similar results.
(TIF)

Figure S4 ISH of vascular genes following Etv2 expression. *Fli1a* (A), *tal1* (B), *erg* (C), and *kdr* (D) are all induced in the trunk of embryos 8 h post–heat shock (HS+8 h). Normally these genes are specifically expressed in the vasculature at this time point (control), although *tal1* is more strongly expressed in the blood and neurons in the CNS. Note that *fli1a* and *erg* have almost ubiquitous expression at this time point, while *tal1* and *kdr* are more restricted to ectopic expression in the trunk. Quantification of the number of embryos demonstrating ectopic expression over the number observed is in the bottom right corner of the corresponding high-magnification trunk image for each group. Eight hours post–heat shock was chosen because it is the time when ectopic expression of *kdrl:GFP* was first noted in our transgenic analysis.
(TIF)

Figure S5 ISH of muscle genes following Etv2 expression. *Myog* (A), *myf6* (B), *mylpfa* (C), and *tnnt3a* (D) are all repressed in the trunk of embryos 4 h post–heat shock (HS+4 h). Normally these genes are strongly and specifically expressed in the musculature at this time point (control). Expression of *myog* and *myf6* is almost completely abolished (A, B). *Mylpfa* and *Tnnt3a* are reduced but much less so (C, D). Quantification of the number of embryos demonstrating normal muscle expression over the number observed is in the bottom right corner of the corresponding high-magnification trunk image for each group. Four hours post–heat shock was chosen since it is the peak of heat shock–induced Etv2 expression.
(TIF)

Figure S6 Fli1a and Tal1 overexpression at 24 hpf is not sufficient to induce ectopic *kdrl:GFP* expression at 48 hpf. (A) Heat shock–induced *etv2*, *fli1a*, or *tal1* are all capable of inducing ectopic *kdrl:GFP^+^* cells in the early embryo. *Kdrl:GFP* embryos were injected with the indicated heat shock–inducible transgenes, heat shocked at shield stage, and imaged at 16 somite stage. Nuclear mCherry (NLS-mCherry) was not able to induce ectopic *kdrl:GFP* while *etv2*, *fli1a*, and *tal1* were (bracketed area in lateral view of embryo). The number of embryos exhibiting ectopic GFP expression over the total number observed is represented in the top right corner of each panel. (B) *Kdrl:GFP* transgenic embryos were injected with *hsp70l:etv2*, *hsp70:fli1a*, or *hsp70:tal1* transgenes and heat shocked at 24 hpf. Each transcription factor was labeled with mCherry and expression was confirmed by imaging 3 h post–heat shock (inset). GFP expression was imaged at 48 hpf. Etv2 overexpression resulted in strong ectopic GFP expression, but neither Fli1a nor Tal1 was sufficient for inducing the same response. The ratio in the bottom right corner of each panel represents the GFP positive embryos over the total observed.
(TIF)

Figure S7 Slow muscle fibers do not respond to Etv2 overexpression. Immunostained sections through the trunk of 48 hpf hsp70l:etv2/fli1a:EGFP embryos that were untreated (control) or heat shocked at 24 hpf (HS+24 h). Sections were stained for GFP and slow muscle myosin. Nuclei are stained with DAPI in the mergeDAPI panels. fli1a:EGFP is normally expressed in the intersomitic vessels (ISVs) and axial vessels (AVs) of control sections. No co-staining of GFP and slow muscle myosin was observed (arrows). ROI is the region of interest highlighted by the dashed box in each panel. One section from 20 different embryos was observed for each treatment group with similar results within each group.
(TIF)

Figure S8 Fast muscle–specific *mylpfa:mRFP* is co-expressed with *kdrl:GFP* following overexpression of Etv2. (A) Confocal image of the trunk of a *kdrl:GFP*/*mylpfa:mRFP*/*hsp70l:etv2* triple transgenic embryo heat shocked at 24 hpf and imaged at 12 h post–heat shock. A GFP/mRFP double positive muscle fiber is highlighted by the arrow and *x*-axis and *y*-axis z-plane projections are presented below and to the right of the image respectively. (B) Fast muscle myosin and GFP colocalize in the trunk of *kdrl:GFP/hsp70l:etv2* transgenic fish heat shocked at 24 hpf and imaged at 48 hpf. Red muscle fibers colocalize with GFP (white arrows). A strongly GFP positive cell located where a muscle fiber normally would be is negative for fast muscle myosin (large white arrowhead), suggesting this cell has lost its muscle cell identity.
(TIF)

Figure S9 Etv2 overexpression is toxic to angiogenic sprouts and its maintained expression prevents *kdrl:GFP* expression. (A) Time lapse imaging of an intersegmental vessel angiogenic sprout from Figure 3C showing apparent apoptosis. (B) Multiple heat shocks of *hsp70l:etv2* embryos prevent ectopic *kdrl:GFP* expression. Embryos were initially heat shocked at 24 hpf and then either maintained at normal temperatures or were treated with heat shock every 12 h the indicated number of times. Embryos treated with multiple heat shocks displayed abnormal morphology whose severity correlated with the number of heat shocks, including cardiac edema suggestive of circulatory failure (left column and inset). Control embryos heat shocked four times appeared similar to non–heat shocked embryos, HS(–). When Etv2 expression was maintained for the whole period (4× heat shocks), *kdrl:GFP* was not ectopically induced and was reduced or absent in the normal vasculature (right column).
(TIF)

Figure S10 Lineage tracing of muscle cells demonstrate they are a source of new blood vessels following Etv2 expression. Lineage tracing of fast muscle fibers in *mylpfa:cre-ERt2* injected *kdrl:GFP/hsp70l:etv2/ubi:Switch* fish demonstrates endothelial cells derived from muscle fibers (arrow). The *ubi:Switch* transgene changes from GFP to mCherry following recombination initiated by Cre. The *mylpfa* promoter specifically drives Cre expression in fast muscle fibers. In non–heat shocked embryos (−HS) only mCherry muscle fibers are observed (*n* = 13), while following heat shock mCherry^+^, *kdrl:GFP* ^+^ vessels were observed in 5 out of 15 embryos observed. Note that the *kdrl:GFP* transgene in the vasculature is significantly brighter than the *ubi:Switch* GFP^+^ background. All embryos were treated with hydroxytamoxifen (5 µM) immediately after heat shock or the equivalent time for non–heat shocked controls.
(TIF)

Figure S11 Etv2 cell autonomously initiates transdifferentiation of muscle cells. Blastula cell transplantation was performed from triple transgenic, *mylpfa:mRFP/hsp70l:etv2/kdrl:GFP+,* into wild-type embryos. Approximately 10 cells were transplanted per embryo. Transplanted embryos were raised until 22 hpf at which point they were selected for embryos displaying mylpfa:mRFP expression in distinct regions absent in *kdrl:GFP.* These embryos were then either heat shocked or left as no heat shock controls. Embryos were then analyzed for *mylpfa:mRFP/kdrl:GFP* coexpression at 10 h post–heat shock and followed out to 44 h post–heat shock. Two *kdrl:GFP* positive muscle fibers, one still mylpfa:mRFP positive (arrow), under-go transdifferentiation to form functional vessels supporting blood cell flow (see Movie S4).
(TIF)

Figure S12 Activation of Wnt signaling with different doses of CHIR99021 can expand transdifferentiation at 28 hpf. *Hsp70l:etv2*/*kdrl:GFP* embryos were heat shocked at 28 hpf, treated with various doses of CHIR99021, and GFP^+^ myotomes were quantified at 48 hpf. Doses between 5 and 25 µM significantly increased the number of GFP^+^ myotomes but not to the levels seen when heat shock was administered at earlier time points. *t* test, (*) *p*<0.05, (**) *p*<0.01.
(TIF)

Figure S13 Etv2 induces vascular marker gene expression in C2C12 cells but not in other cell types. (A) Induction of endothelial cells marker expression in myoblast cell line C2C12. Fli1, Scl, Kdr, and vascular endothelial cadherin (Vecad) expression levels detected by qPCR were compared between Etsrp/Etv2 transfection and control mCherry transfection. Fold of induction compared to nontransfected cells is shown. (B) PCR detection of vascular marker expression in three tumor cell lines that were transfected with Etv2. No induction of the listed endothelial cell markers was detected.
(TIF)

Movie S1 *Kdrl:gfp/mylpfa:mRFP/hsp70l:etv2* non–heat shocked control.
(MOV)

Movie S2 *Kdrl:gfp/mylpfa:mRFP/hsp70l:etv2* heat shocked at 24 hpf and imaged from +8 h to +22 h.
(MOV)

Movie S3 Timelapse imaging of a single fast muscle fiber becoming kdrl:GFP positive and changing morphology. This movie corresponds to the right panels of Figure 3C.
(MOV)

Movie S4 Blood flow through muscle-derived endothelial cells in a chimeric embryo. Video corresponds to the region highlighted in Figure S11. The *kdrl:GFP^+^* cells in the upper left are muscle derived and support blood circulation.
(MOV)

Table S1 Gene-specific primers for qPCR.
(DOCX)

## Abbreviations

EGFP: enhanced green fluorescent protein
EYFP: enhanced yellow fluorescent protein
GFP: green fluorescent protein
hpf: hours postfertilization
*hsp70l:etv2*: *hsp70l:etv2-mCherry*
ISH: in situ hybridization
MO: morpholino
mRFP: monomeric red fluorescent protein
OE: overexpression
qPCR: quantitative reverse transcription polymerase chain reaction
VEGF: vascular endothelial growth factor
