# Supplementary figures and images for: Transdifferentiation of Fast Skeletal Muscle Into Functional Endothelium in Vivo by Transcription Factor Etv2
Source: PLoS Biol. 2013 Jun 18;11(6):e1001590. doi: 10.1371/journal.pbio.1001590 (PMC3708712; doi:10.1371/journal.pbio.1001590)

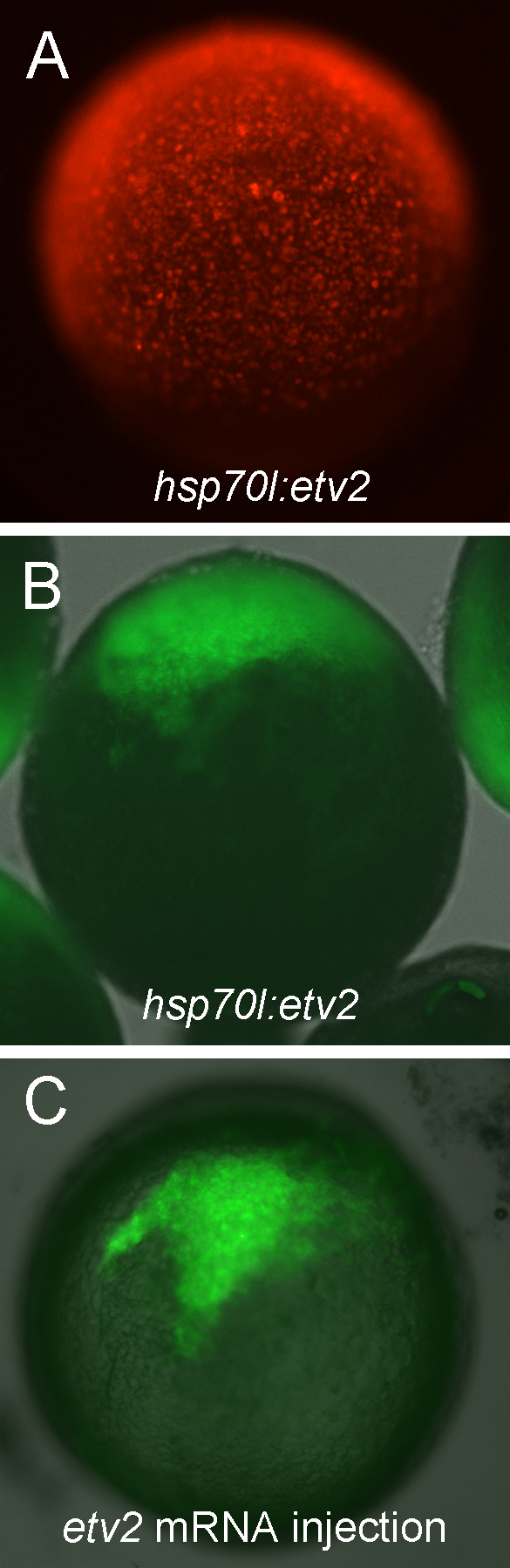

Supplement: Figure S1 — Hsp70l:etv2 is as effective as etv2 mRNA injection at inducing kdrl:GFP expression. (A) A shield stage embryo that was heat shocked at dome stage and imaged for mCherry expression. Nuclear-localized Etv2-mCherry is visible. (B) Heat shock of hsp70l:etv2-mCherry transgenic embryos at dome stage induces kdrl:GFP expression at tailbud stage similar to that seen with mRNA injection. (C) mRNA injection of etv2 induces kdrl:GFP expression at tailbud stage. The embryos overexpressing Etv2 in (C) and (D) gastrulated abnormally due to overexpressed Etv2. (TIF) [file pbio.1001590.s001.tif]

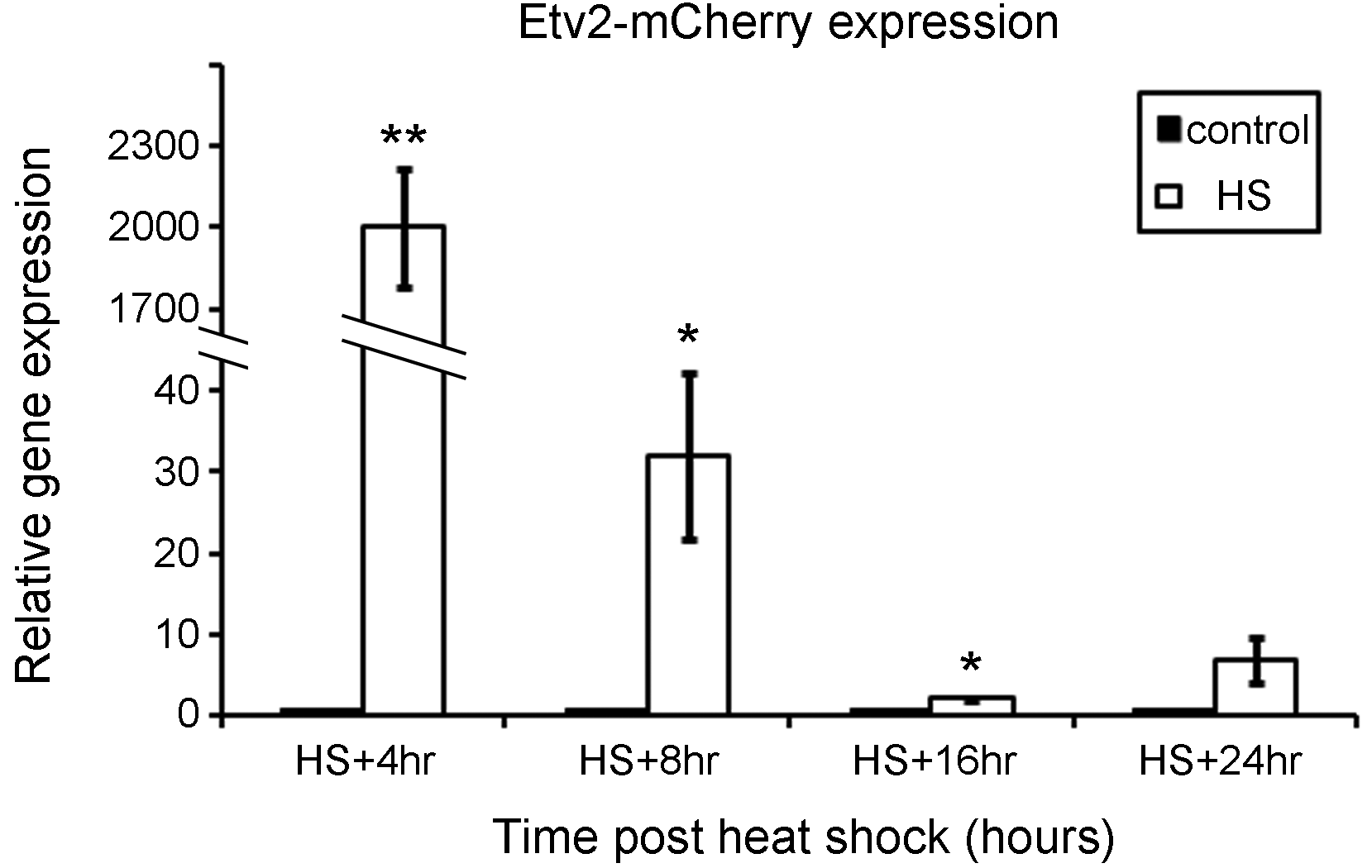

Supplement: Figure S2 — qRT-PCR quantification of Etv2-mCherry following heat shock. Etv2-mCherry expression is very high 4 h post–heat shock but quickly decreases to levels similar to control by 24 h post–heat shock. This time course of expression is very similar to that observed by nuclear Etv2-mCherry fluorescence. (TIF) [file pbio.1001590.s002.tif]

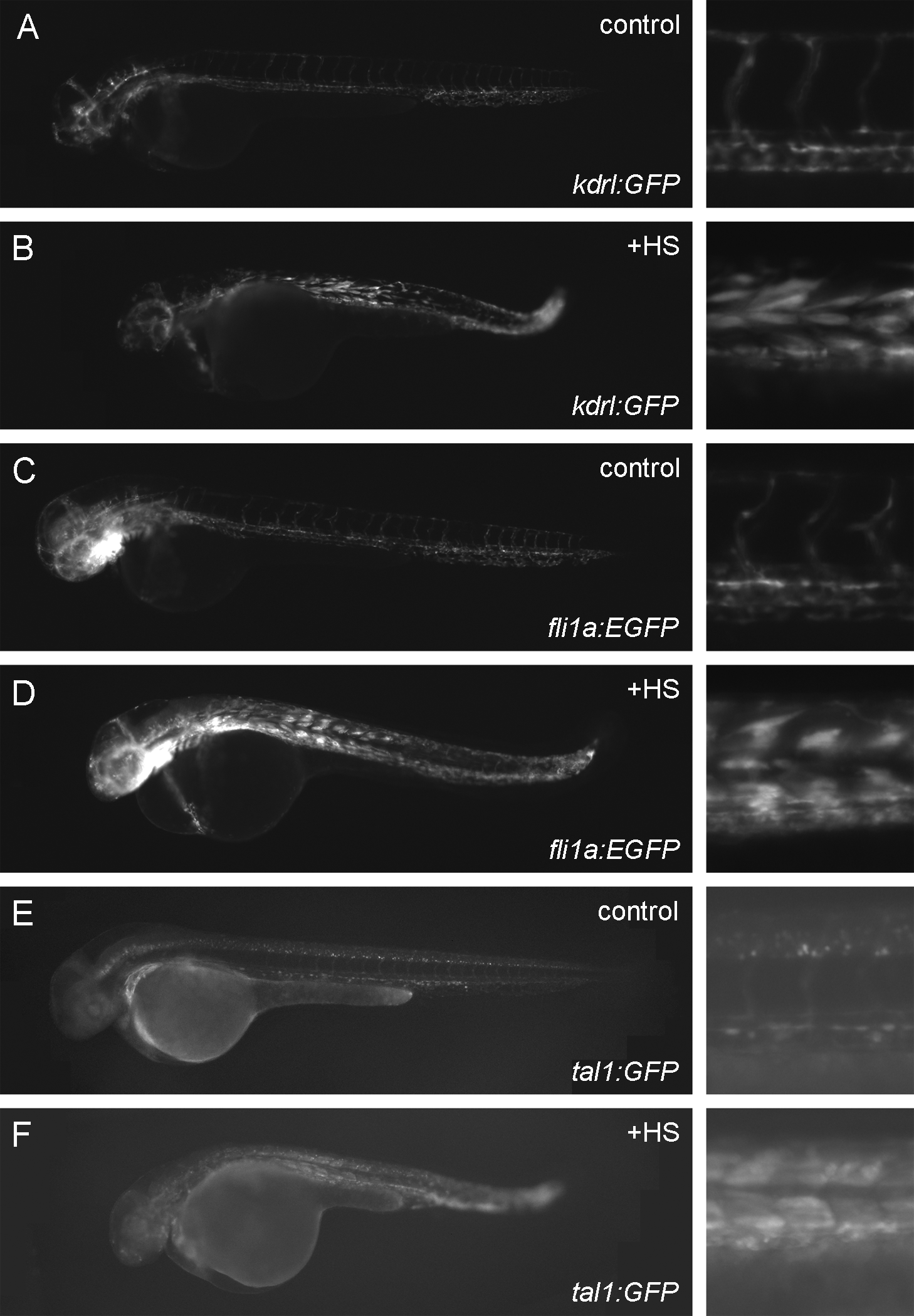

Supplement: Figure S3 — Kdrl:GFP, fli1a:EGFP and tal1:GFP are all induced in the trunk of hsp70l:etv2 embryos following heat shock. Hsp70l:etv2 was crossed into kdrl:GFP (A, B), fli1a:EGFP (C, D), or tal1:GFP (E, F), and the resulting embryos were either left at control temperature or were heat shocked at 22 hpf and then imaged at 48 hpf. Control embryos never exhibited ectopic GFP expression in any group. Heat-shocked embryos (+HS) always exhibited ectopic GFP expression in the trunk (right column is high magnification image of the trunk corresponding to the adjacent embryo in the left column), although tal1:GFP was significantly weaker than the other two transgenes. At least 20 embryos were observed for each treatment with similar results. (TIF) [file pbio.1001590.s003.tif]

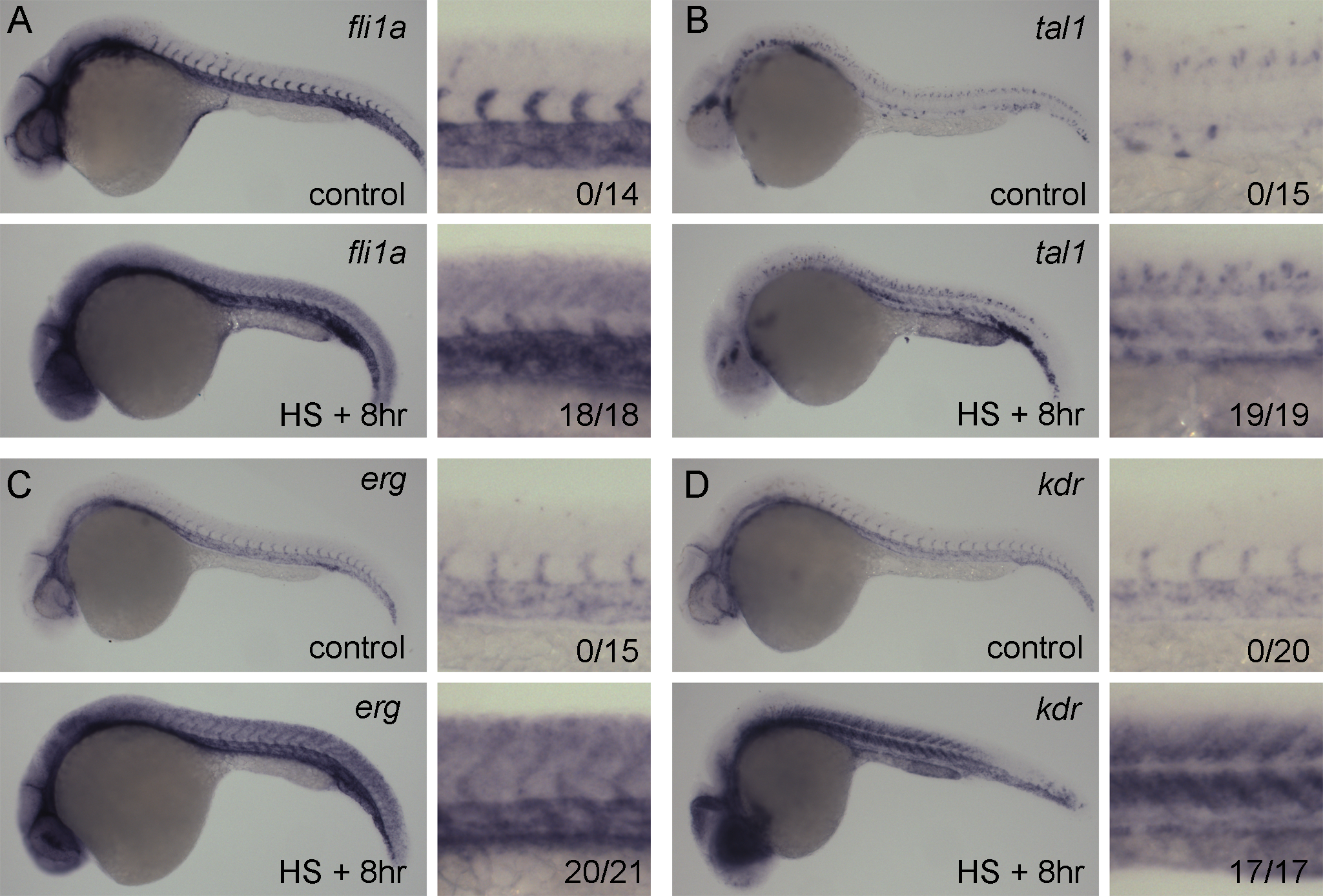

Supplement: Figure S4 — ISH of vascular genes following Etv2 expression. Fli1a (A), tal1 (B), erg (C), and kdr (D) are all induced in the trunk of embryos 8 h post–heat shock (HS+8 h). Normally these genes are specifically expressed in the vasculature at this time point (control), although tal1 is more strongly expressed in the blood and neurons in the CNS. Note that fli1a and erg have almost ubiquitous expression at this time point, while tal1 and kdr are more restricted to ectopic expression in the trunk. Quantification of the number of embryos demonstrating ectopic expression over the number observed is in the bottom right corner of the corresponding high-magnification trunk image for each group. Eight hours post–heat shock was chosen because it is the time when ectopic expression of kdrl:GFP was first noted in our transgenic analysis. (TIF) [file pbio.1001590.s004.tif]

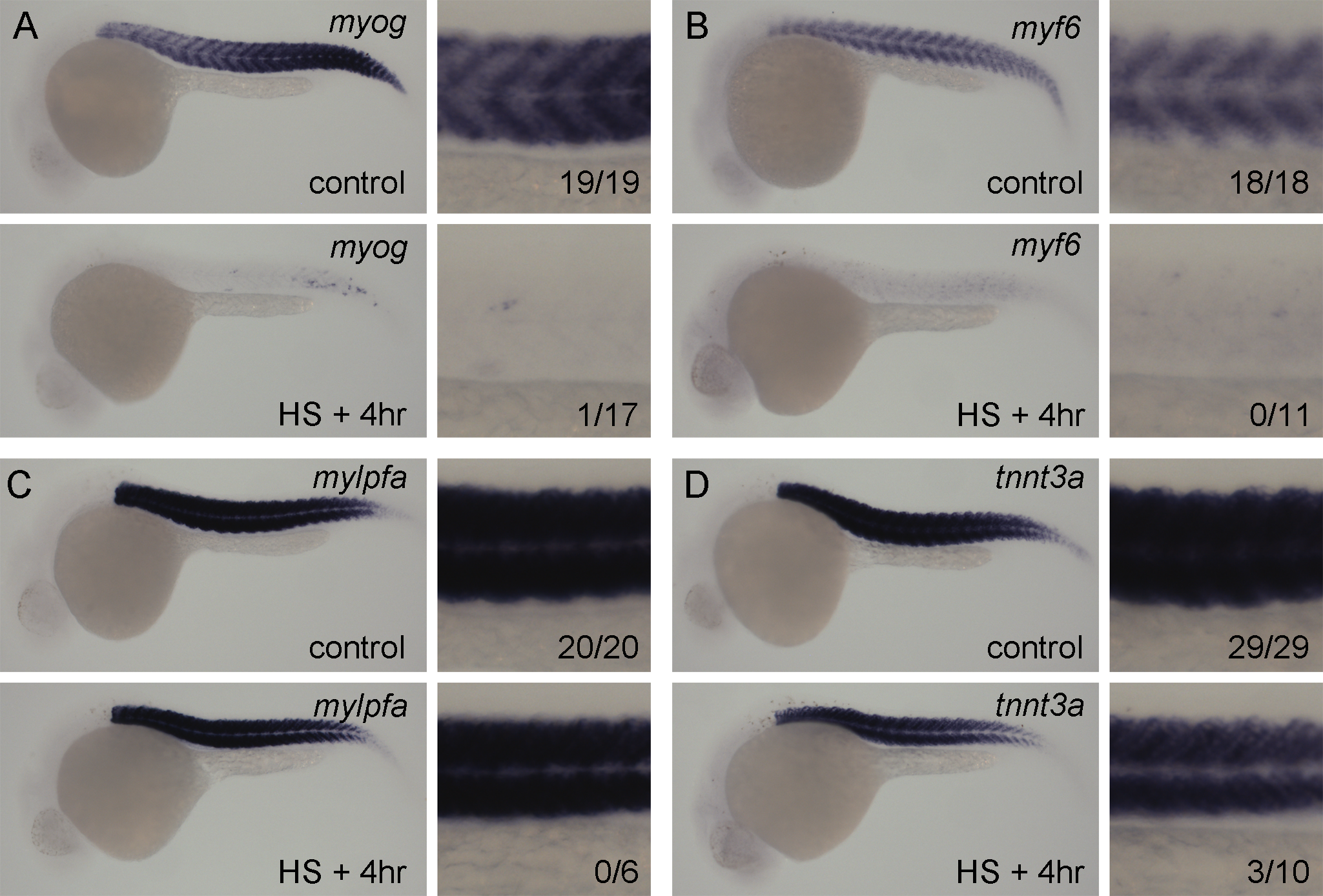

Supplement: Figure S5 — ISH of muscle genes following Etv2 expression. Myog (A), myf6 (B), mylpfa (C), and tnnt3a (D) are all repressed in the trunk of embryos 4 h post–heat shock (HS+4 h). Normally these genes are strongly and specifically expressed in the musculature at this time point (control). Expression of myog and myf6 is almost completely abolished (A, B). Mylpfa and Tnnt3a are reduced but much less so (C, D). Quantification of the number of embryos demonstrating normal muscle expression over the number observed is in the bottom right corner of the corresponding high-magnification trunk image for each group. Four hours post–heat shock was chosen since it is the peak of heat shock–induced Etv2 expression. (TIF) [file pbio.1001590.s005.tif]

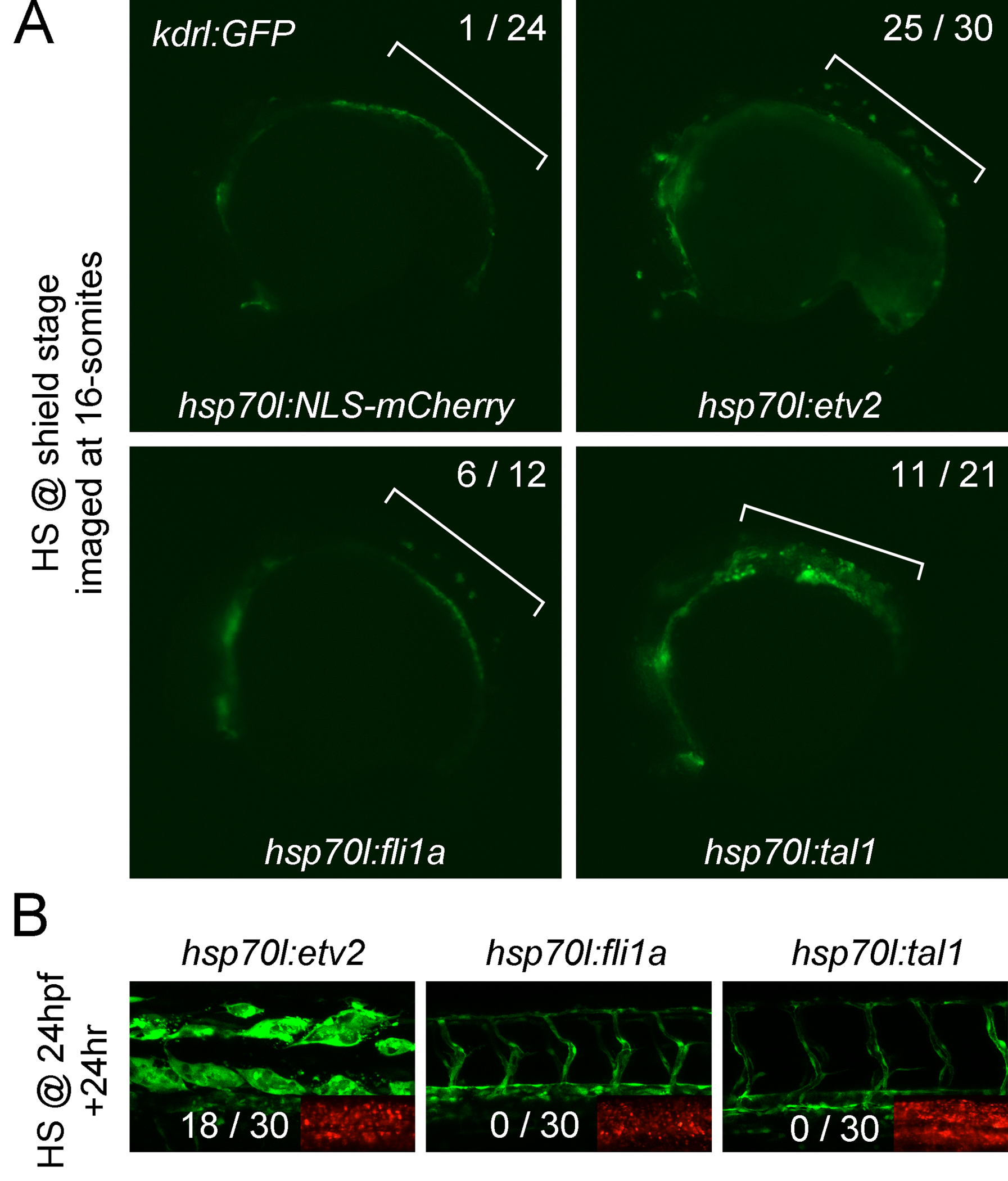

Supplement: Figure S6 — Fli1a and Tal1 overexpression at 24 hpf is not sufficient to induce ectopic kdrl:GFP expression at 48 hpf. (A) Heat shock–induced etv2, fli1a, or tal1 are all capable of inducing ectopic kdrl:GFP+ cells in the early embryo. Kdrl:GFP embryos were injected with the indicated heat shock–inducible transgenes, heat shocked at shield stage, and imaged at 16 somite stage. Nuclear mCherry (NLS-mCherry) was not able to induce ectopic kdrl:GFP while etv2, fli1a, and tal1 were (bracketed area in lateral view of embryo). The number of embryos exhibiting ectopic GFP expression over the total number observed is represented in the top right corner of each panel. (B) Kdrl:GFP transgenic embryos were injected with hsp70l:etv2, hsp70:fli1a, or hsp70:tal1 transgenes and heat shocked at 24 hpf. Each transcription factor was labeled with mCherry and expression was confirmed by imaging 3 h post–heat shock (inset). GFP expression was imaged at 48 hpf. Etv2 overexpression resulted in strong ectopic GFP expression, but neither Fli1a nor Tal1 was sufficient for inducing the same response. The ratio in the bottom right corner of each panel represents the GFP positive embryos over the total observed. (TIF) [file pbio.1001590.s006.tif]

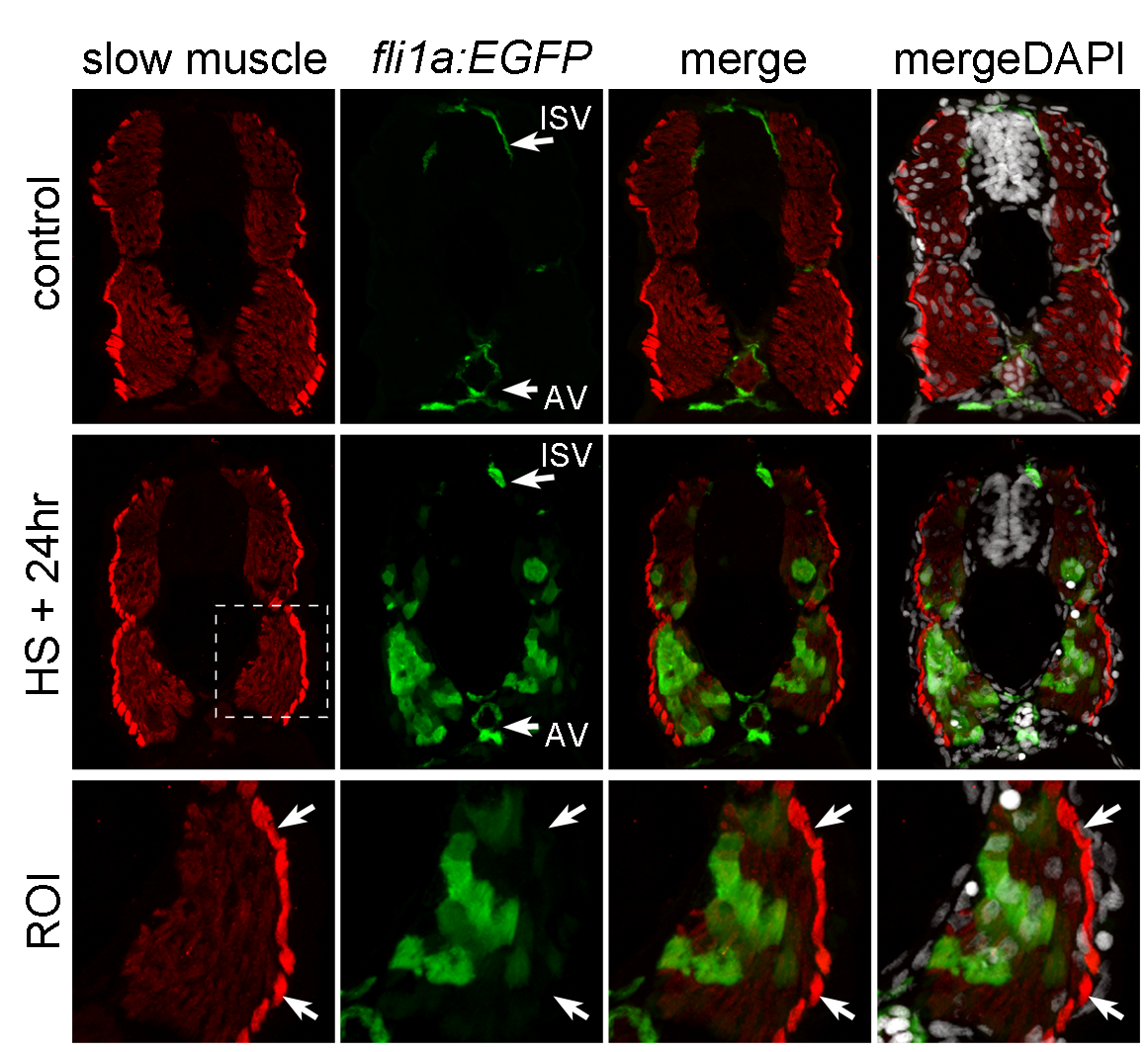

Supplement: Figure S7 — Slow muscle fibers do not respond to Etv2 overexpression. Immunostained sections through the trunk of 48 hpf hsp70l:etv2/fli1a:EGFP embryos that were untreated (control) or heat shocked at 24 hpf (HS+24 h). Sections were stained for GFP and slow muscle myosin. Nuclei are stained with DAPI in the mergeDAPI panels. fli1a:EGFP is normally expressed in the intersomitic vessels (ISVs) and axial vessels (AVs) of control sections. No co-staining of GFP and slow muscle myosin was observed (arrows). ROI is the region of interest highlighted by the dashed box in each panel. One section from 20 different embryos was observed for each treatment group with similar results within each group. (TIF) [file pbio.1001590.s007.tif]

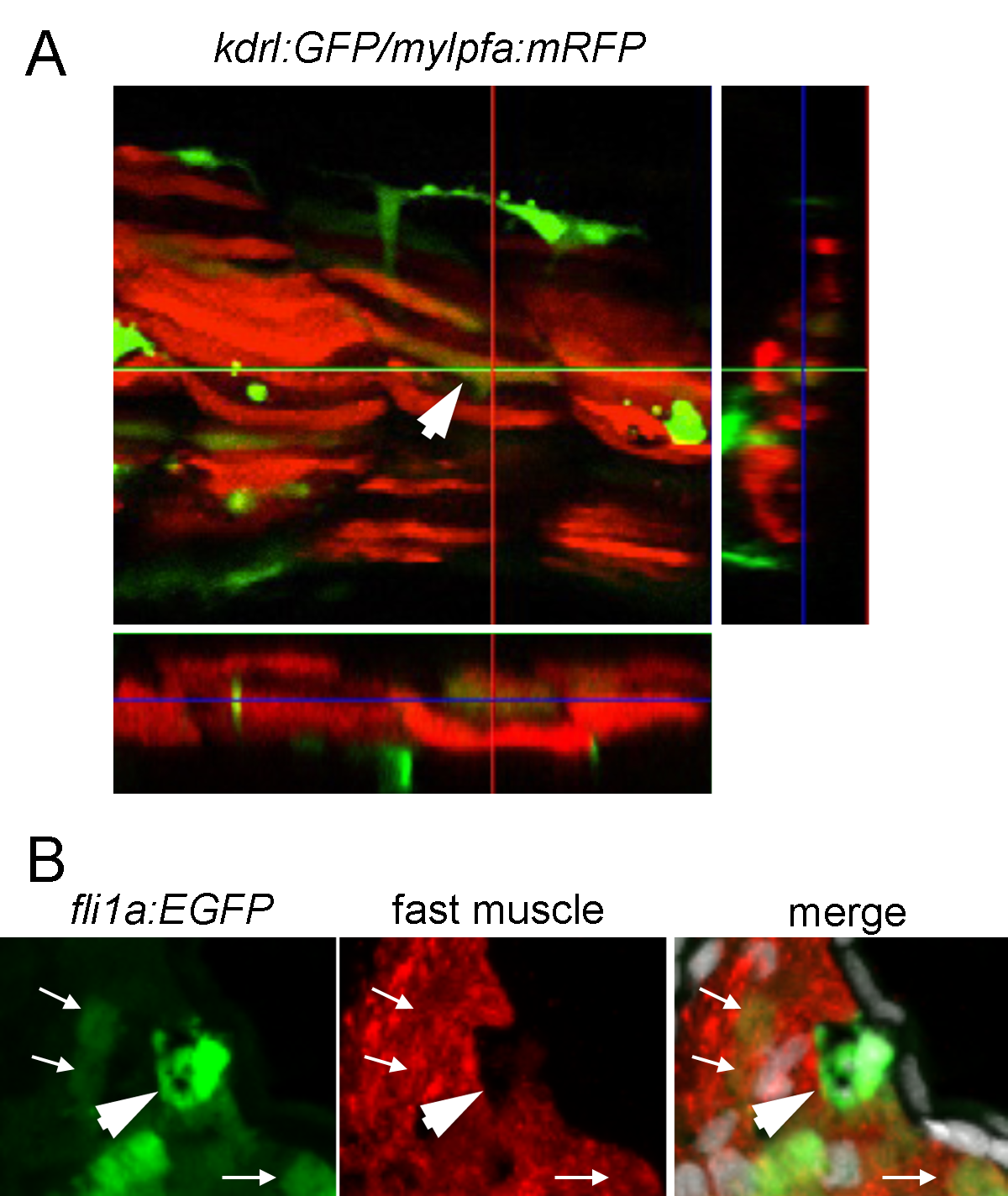

Supplement: Figure S8 — Fast muscle–specific mylpfa:mRFP is co-expressed with kdrl:GFP following overexpression of Etv2. (A) Confocal image of the trunk of a kdrl:GFP/mylpfa:mRFP/hsp70l:etv2 triple transgenic embryo heat shocked at 24 hpf and imaged at 12 h post–heat shock. A GFP/mRFP double positive muscle fiber is highlighted by the arrow and x-axis and y-axis z-plane projections are presented below and to the right of the image respectively. (B) Fast muscle myosin and GFP colocalize in the trunk of kdrl:GFP/hsp70l:etv2 transgenic fish heat shocked at 24 hpf and imaged at 48 hpf. Red muscle fibers colocalize with GFP (white arrows). A strongly GFP positive cell located where a muscle fiber normally would be is negative for fast muscle myosin (large white arrowhead), suggesting this cell has lost its muscle cell identity. (TIF) [file pbio.1001590.s008.tif]

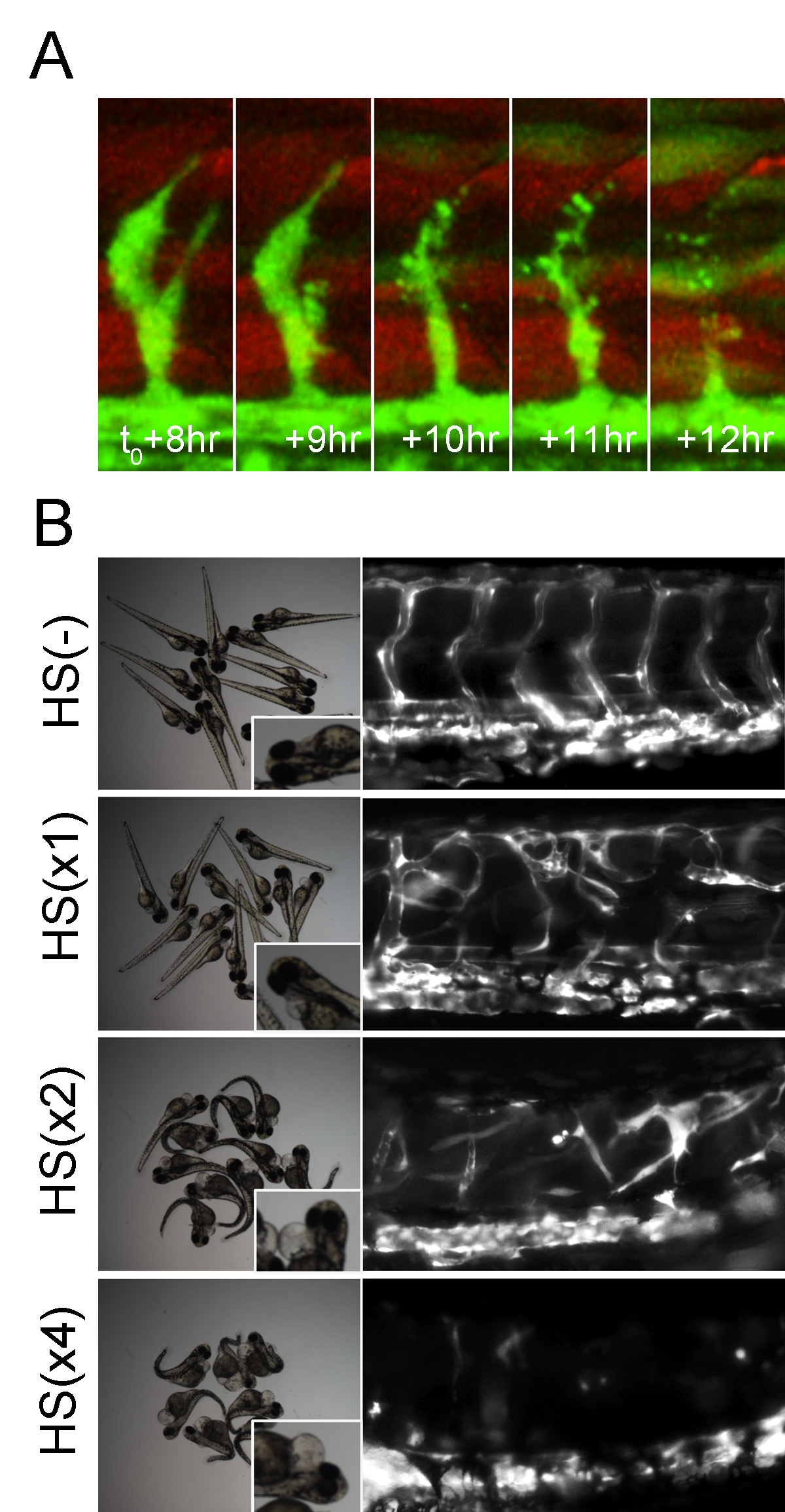

Supplement: Figure S9 — Etv2 overexpression is toxic to angiogenic sprouts and its maintained expression prevents kdrl:GFP expression. (A) Time lapse imaging of an intersegmental vessel angiogenic sprout from Figure 3C showing apparent apoptosis. (B) Multiple heat shocks of hsp70l:etv2 embryos prevent ectopic kdrl:GFP expression. Embryos were initially heat shocked at 24 hpf and then either maintained at normal temperatures or were treated with heat shock every 12 h the indicated number of times. Embryos treated with multiple heat shocks displayed abnormal morphology whose severity correlated with the number of heat shocks, including cardiac edema suggestive of circulatory failure (left column and inset). Control embryos heat shocked four times appeared similar to non–heat shocked embryos, HS(–). When Etv2 expression was maintained for the whole period (4× heat shocks), kdrl:GFP was not ectopically induced and was reduced or absent in the normal vasculature (right column). (TIF) [file pbio.1001590.s009.tif]

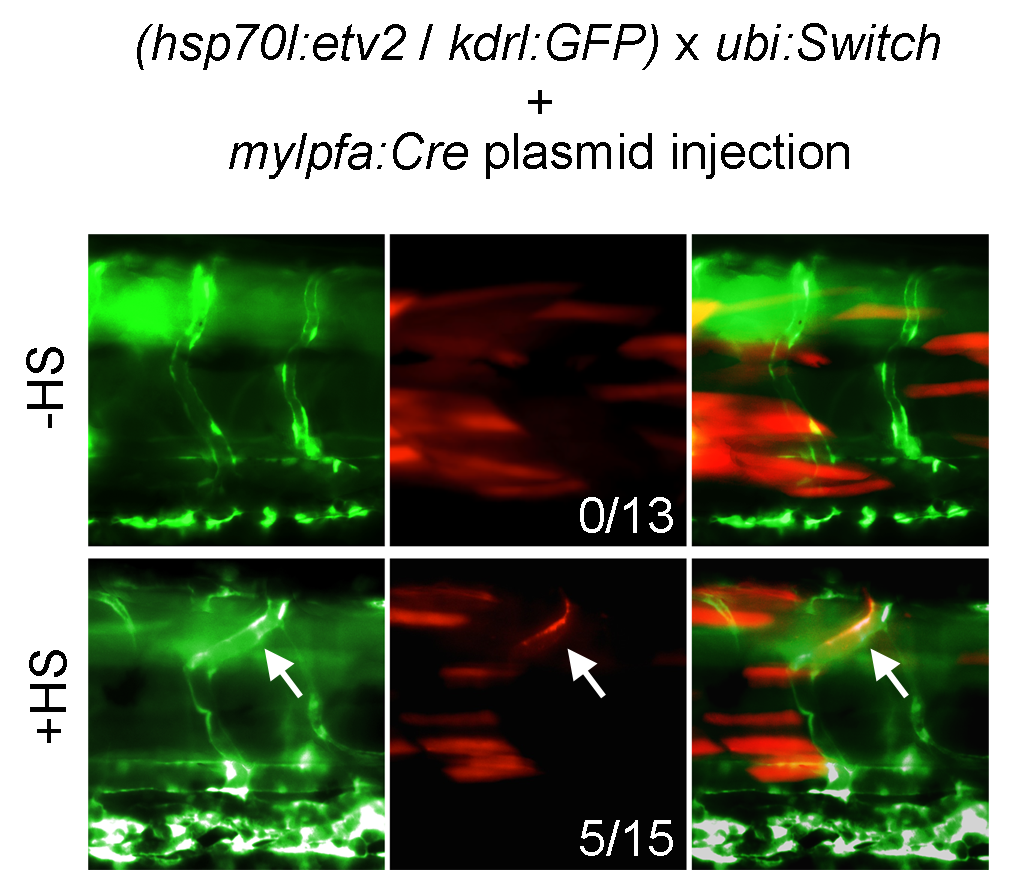

Supplement: Figure S10 — Lineage tracing of muscle cells demonstrate they are a source of new blood vessels following Etv2 expression. Lineage tracing of fast muscle fibers in mylpfa:cre-ERt2 injected kdrl:GFP/hsp70l:etv2/ubi:Switch fish demonstrates endothelial cells derived from muscle fibers (arrow). The ubi:Switch transgene changes from GFP to mCherry following recombination initiated by Cre. The mylpfa promoter specifically drives Cre expression in fast muscle fibers. In non–heat shocked embryos (−HS) only mCherry muscle fibers are observed (n = 13), while following heat shock mCherry+, kdrl:GFP + vessels were observed in 5 out of 15 embryos observed. Note that the kdrl:GFP transgene in the vasculature is significantly brighter than the ubi:Switch GFP+ background. All embryos were treated with hydroxytamoxifen (5 µM) immediately after heat shock or the equivalent time for non–heat shocked controls. (TIF) [file pbio.1001590.s010.tif]

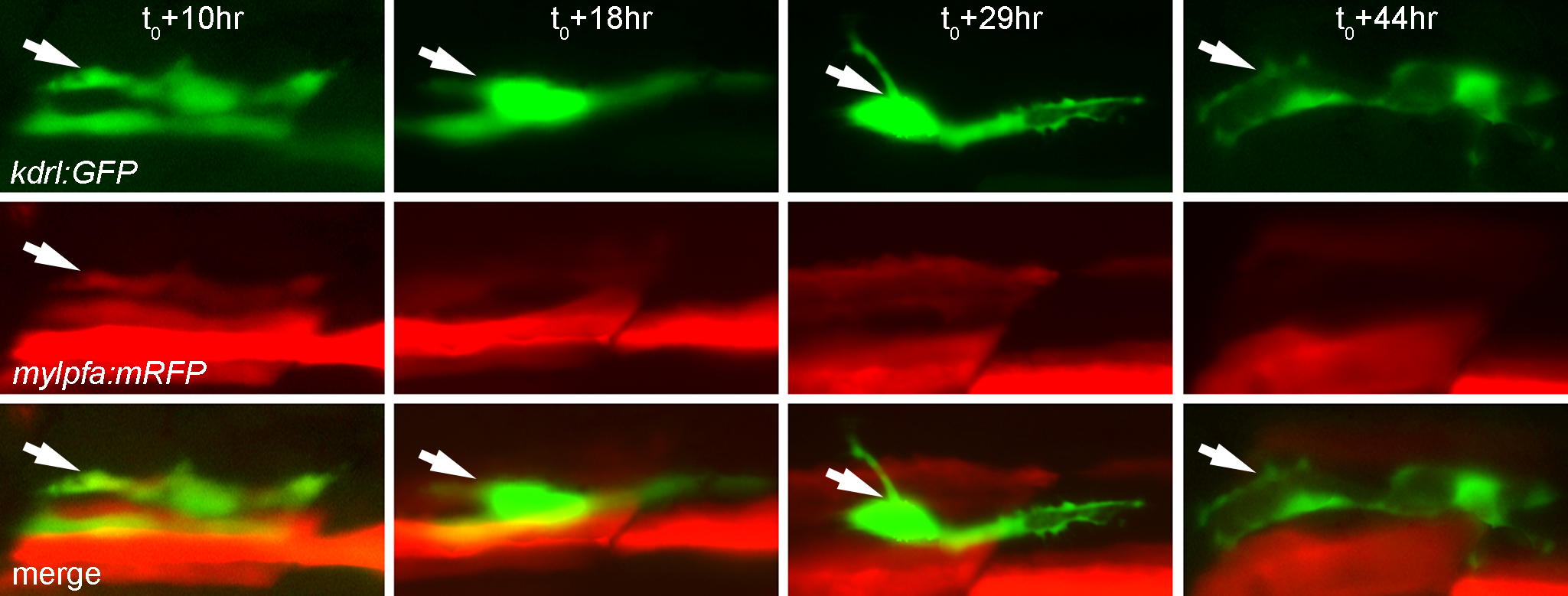

Supplement: Figure S11 — Etv2 cell autonomously initiates transdifferentiation of muscle cells. Blastula cell transplantation was performed from triple transgenic, mylpfa:mRFP/hsp70l:etv2/kdrl:GFP+, into wild-type embryos. Approximately 10 cells were transplanted per embryo. Transplanted embryos were raised until 22 hpf at which point they were selected for embryos displaying mylpfa:mRFP expression in distinct regions absent in kdrl:GFP. These embryos were then either heat shocked or left as no heat shock controls. Embryos were then analyzed for mylpfa:mRFP/kdrl:GFP coexpression at 10 h post–heat shock and followed out to 44 h post–heat shock. Two kdrl:GFP positive muscle fibers, one still mylpfa:mRFP positive (arrow), under-go transdifferentiation to form functional vessels supporting blood cell flow (see Movie S4). (TIF) [file pbio.1001590.s011.tif]

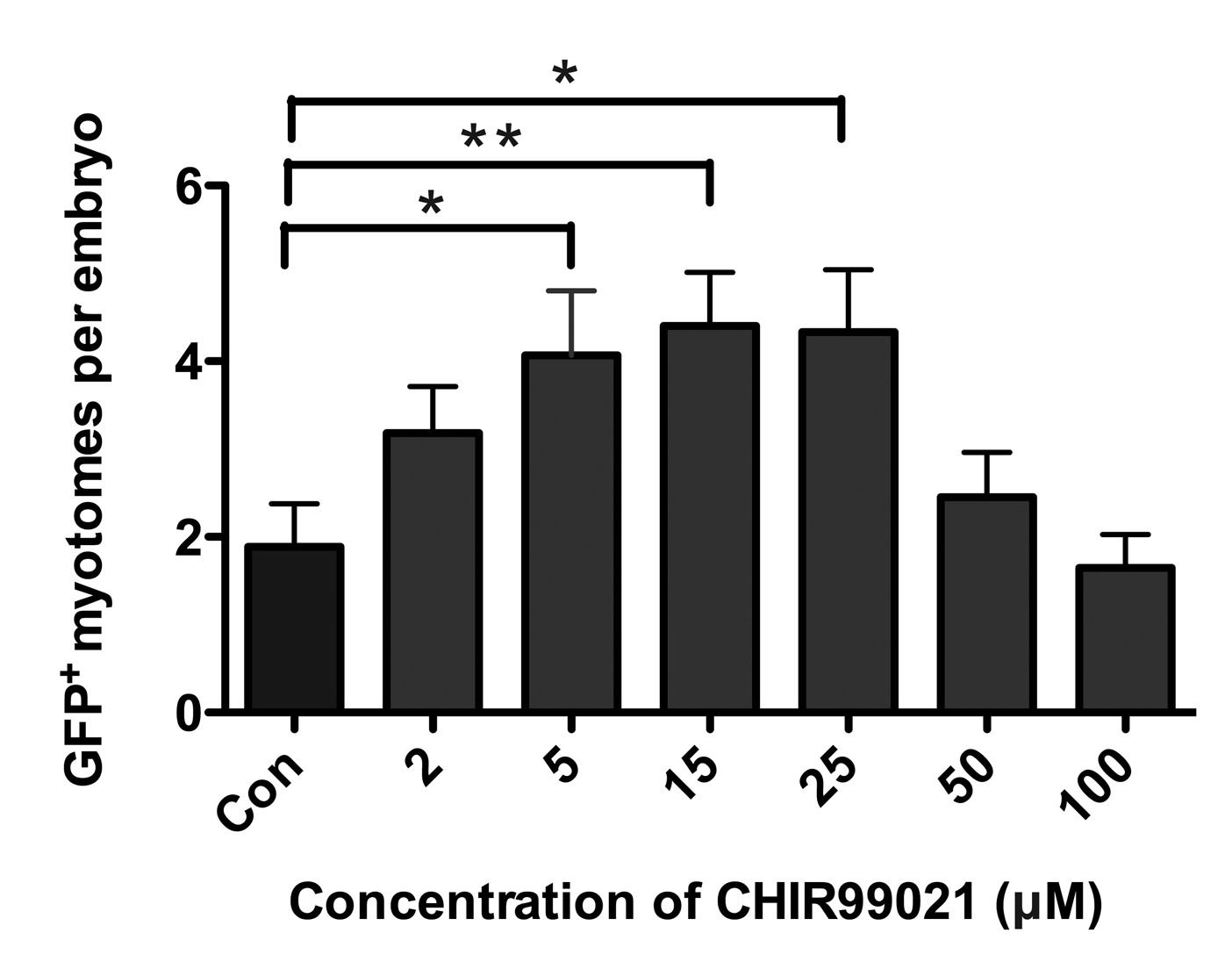

Supplement: Figure S12 — Activation of Wnt signaling with different doses of CHIR99021 can expand transdifferentiation at 28 hpf. Hsp70l:etv2/kdrl:GFP embryos were heat shocked at 28 hpf, treated with various doses of CHIR99021, and GFP+ myotomes were quantified at 48 hpf. Doses between 5 and 25 µM significantly increased the number of GFP+ myotomes but not to the levels seen when heat shock was administered at earlier time points. t test, (*) p<0.05, (**) p<0.01. (TIF) [file pbio.1001590.s012.tif]

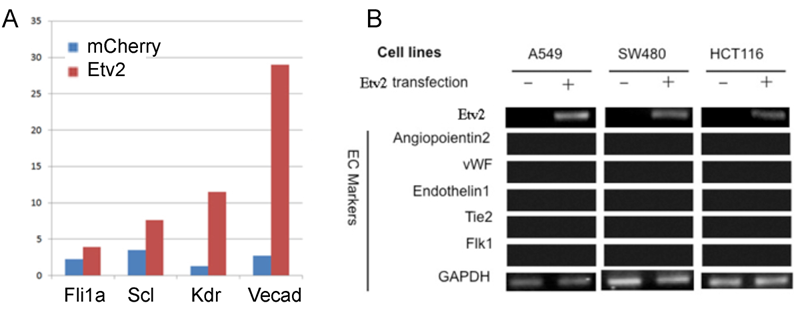

Supplement: Figure S13 — Etv2 induces vascular marker gene expression in C2C12 cells but not in other cell types. (A) Induction of endothelial cells marker expression in myoblast cell line C2C12. Fli1, Scl, Kdr, and vascular endothelial cadherin (Vecad) expression levels detected by qPCR were compared between Etsrp/Etv2 transfection and control mCherry transfection. Fold of induction compared to nontransfected cells is shown. (B) PCR detection of vascular marker expression in three tumor cell lines that were transfected with Etv2. No induction of the listed endothelial cell markers was detected. (TIF) [file pbio.1001590.s013.tif]
