# Supplementary material for: Transdifferentiation of Fast Skeletal Muscle Into Functional Endothelium in Vivo by Transcription Factor Etv2
Source: PLoS Biol. 2013 Jun 18;11(6):e1001590. doi: 10.1371/journal.pbio.1001590 (PMC3708712; doi:10.1371/journal.pbio.1001590)
Supplement: Table S1 — Gene-specific primers for qPCR. (DOCX) [file pbio.1001590.s018.docx]

| Zf βactin1 F | TGTTTTCCCCTCCATTGTTG |
| --- | --- |
| Zf βactin1 R | ACATACATGGCAGGGGTGTT |
| Zf kdrl F | CGCAAAGGAGACGCTAGACT |
| Zf kdrl R | AGATGGTTCTCCATGCCAGC |
| Zf kdr F | CTGCACCACAGTAGCAGTGA |
| Zf kdr R | GCGTCCGTTTCTTGTAGGGA |
| Zf fli1a F | TCGTCCTCAGCCAGATCC |
| Zf fli1a R | TGGTTCCTTCCCAGGTGA |
| Zf tal1 F | GGAGATGCGGAACAGTATGG |
| Zf tal1 R | GAAGGCACCGTTCACATTCT |
| Zf erg F | AACCAACGGCGAGTTCAAGA |
| Zf erg R | CCACCGCAGGGTACTTGTAG |
| Zf myod1 F | CACACCAAATGCTGACGCAC |
| Zf myod1 R | ATCCCTCATGCGGAGAACAC |
| Zf myog F | GCTCCACATACTGGGGTGTC |
| Zf myog R | GCCTCTGTTCCCGTTATGCT |
| Zf myf6 F | AGACGGTGCCTAATCCGAAC |
| Zf myf6 R | TCCAACACGGCTCCTTCTCT |
| Zf mylpfa F | GTCTCGACATGGCACCCAAG |
| Zf mylpfa R | GAGGCCAACACGTCCCTAAG |
| Zf tnnt3a F | TGGCTGAGAGACGCAAACAA |
| Zf tnnt3a R | TGCGCAGTGTAGTAACCTCG |

Table S1. qRT-PCR primers.
